# Transcriptomic characterization and curation of candidate neuropeptides regulating reproduction in the eyestalk ganglia of the Australian crayfish, *Cherax quadricarinatus*

**DOI:** 10.1038/srep38658

**Published:** 2016-12-07

**Authors:** Tuan Viet Nguyen, Scott F. Cummins, Abigail Elizur, Tomer Ventura

**Affiliations:** 1 GeneCology Research Centre, Faculty of Science, Health, Education and Engineering, University of the Sunshine Coast, Sunshine Coast, Queensland, Australia

## Abstract

The Australian redclaw crayfish (*Cherax quadricarinatus*) has recently received attention as an emerging candidate for sustainable aquaculture production in Australia and worldwide. More importantly, *C. quadricarinatus* serves as a good model organism for the commercially important group of decapod crustaceans as it is distributed worldwide, easy to maintain in the laboratory and its reproductive cycle has been well documented. In order to better understand the key reproduction and development regulating mechanisms in decapod crustaceans, the molecular toolkit available for model organisms such as *C. quadricarinatus* must be expanded. However, there has been no study undertaken to establish the *C. quadricarinatus* neuropeptidome. Here we report a comprehensive study of the neuropeptide genes expressed in the eyestalk in the Australian crayfish *C. quadricarinatus*. We characterised 53 putative neuropeptide-encoding transcripts based on key features of neuropeptides as characterised in other species. Of those, 14 neuropeptides implicated in reproduction regulation were chosen for assessment of their tissue distribution using RT-PCR. Further insights are discussed in relation to current knowledge of neuropeptides in other species and potential follow up studies. Overall, the resulting data lays the foundation for future gene-based neuroendocrinology studies in *C. quadricarinatus*.

Neuropeptides play an important role in the regulation of both vertebrate and invertebrate reproduction^1^,^2^,^3^. By binding to receptors in target tissues, neuropeptides elicit a number of downstream cascades including changes in secondary messengers, phosphorylation and eventually altered transcription activity which leads to a biological response^4^. *In silico* neuropeptidome research aimed at understanding different physiological aspects of crustacean biology has been extensively studied in several crustacean species, including *Scylla paramamosain*^5^, *Macrobrachium rosenbergii*^6^, *Procambarus clarkii*^7^, *Homarus americanus* and *Sagmariasus verreauxi*^8^,^9^.

The crustacean eyestalk (together with the supra-oesophageal and thoracic ganglia), is known to be the primary source of expression and secretion of key factors which regulate many aspects of crustacean behaviour and physiology, including growth and reproduction^10^,^11^,^12^. These biological processes operate under the multifaceted control of biogenic amines, neuropeptides, hormones and lipid metabolites such as methyl farnesoate and ecdysteriods^13^. Crustacean neuropeptide research has focused on a number of key factors that are related to reproduction and sexual development. For instance, the immunoreactivity of antibodies raised against gonadotropin releasing hormone (GnRH) in the central nervous system of the giant freshwater prawn *M. rosenbergii* suggests a vertebrate-like reproductive system in crustaceans^14^. Similarly, a GnRH-like peptide was found in the white leg shrimp *Litopenaeus vannamei*^15^, and the primary structure of GnRH was then reported by mass spectral analysis in the red swamp crayfish *P. clarkii*^16^. In addition, a recently discovered crustacean female sex hormone (CFSH) in the blue crab *Callinectes sapidus*^17^ has been implicated in female sexual maturation, leading to identification of orthologs in the crayfish *P. clarkii*^7^ and the Eastern spiny lobster *S. verreauxi*^9^ In addition, research has been focused into the crustacean androgenic gland specific insulin-like hormone^18^,^19^,^20^,^21^,^22^, work which was recently supplemented with the characterization of its receptor^23^,^24^.

In parallel, a well-known candidate for reproduction studies is the Crustacean Hyperglycemic Hormone (CHH) superfamily, which includes CHH itself, as well as Molt Inhibiting Hormone (MIH), Gonad Inhibiting Hormone (GIH, also referred to as Vitellogenesis Inhibiting Hormone (VIH)), and Ion transport protein (ITP) [reviewed by Webster, *et al*.^25^, Chan, *et al*.^13^, and Subramoniam^26^]. Still, it is important to note that in spite of the known function of some members in the CHH superfamily, their identity and functionality is vastly uncharacterised^13^. Lastly, there are numerous neuropeptides that have previously been demonstrated to have other functions (for example, cardio-activity, osmoregulation, pigmentation, stress response) but then later were assigned novel roles in reproduction and/or sexual development^27^,^28^,^29^,^30^. These findings highlight that while our understanding of the molecular mechanisms of neuropeptides involved in crustacean reproduction processes is quite advanced, it is yet still far from being complete.

In the past, classical peptidomics studies used liquid chromatography and/or mass spectrometry to detect the neuropeptides present in the species^31^. However, with the recent advancement in next generation sequencing, bioinformatics analyses platforms as well as the availability of transcriptome databases, the pace at which neuropeptides are discovered has accelerated considerably. Within a short timeframe, a list of putative neuropeptides in a vast number of non-model crustacean species has been generated including: A-type allatostatin (AST-A), B-type allatostatin (AST-B), C-type allatostatin (AST-C), bursicon, CHH, crustacean cardioactive peptide (CCAP), diuretic hormone 31 (DH31), diuretic hormone 44 (DH44), eclosion hormone (EH), myosuppressin, orcokinin, short neuropeptide F (SPF), neuroparsin (NP), pyrokinin, pigment-dispersing hormone (PDH), red pigment-concentrating hormone (RPCH), short neuropeptide F (sNPF), SIFamide (SIF), sulfakinin and tachykinin (TK). This approach effectively characterised the neuropeptidome for a number of crustacean species that lack genomic information^5^,^6^,^7^,^8^,^9^,^32^. Still, application of this information for the proper control of reproduction is in its infancy, primarily due to the gaps between *in silico* and *in vivo* functional experiments.

The Australian redclaw crayfish *Cherax quadricarinatus*, has recently received attention as an emerging candidate for sustainable aquaculture production in Australia and abroad^33^. FAO predicts that *C. quadricarinatus* would be an attractive freshwater species due to the relative ease of farming and distinct advantages over mariculture^34^ provided proper guidelines and sustainable management practices are implemented. Moreover, *C. quadricarinatus* serves as a good model organism for the commercially important group of decapod crustaceans; it is hardy, endures a high range of conditions, easy to maintain in the laboratory and its reproductive cycle has been well documented^35^, with a unique feature of intersex individuals which occur naturally in the population^36^,^37^,^38^,^39^. The molecular toolbox available for *C. quadricarinatus* was recently supplemented with several transcriptomes^40^,^41^ however, no study has been undertaken to establish the *C. quadricarinatus* neuropeptidome.

This study reports the *in silico* mining and characterization of 53 predicted neuropeptide-encoding genes in *C. quadricarinatus*. Fourteen out of these neuropeptide genes, which were previously linked with reproduction, were screened for tissue spatial expression. Alongside, based on knowledge gained from other crustacean neuropeptide studies, we also highlighted several key candidates for future gene-based studies. Utilizing this molecular toolkit could enhance the aquaculture practice of *C. quadricarinatus* and other crustaceans through comparative studies.

## Materials and Methods

### Ethical statement

*C. quadricarinatus* are not endangered or protected in Australia where sampling occurred. Animal use and care protocols were approved by the GeneCology Research Centre. No special sampling permission was required.

### Animals

Mature individuals of the Australian red claw crayfish *C. quadricarinatus*, ranging in size from 35 to 60 g, were purchased from a local farmer in Queensland, Australia. Prior to dissections, individuals were chilled in ice-cold water for 20 minutes to minimize handling stress. For the eyestalk transcriptome, eyestalks from 15 males and 15 females were pooled for each gender separately. For RT-PCR analysis, 10 female individuals, whose tissues were retrieved, were individually weighed and their carapace lengths measured, as well as the weight of their hepatopancreas and ovary. Crayfish tissues including eyestalk, brain and thoracic ganglia (combined), ovary, hepatopancreas, heart, abdomen muscle and antennal gland were dissected and stored in −80 °C until used. Gonadosomatic index was calculated as the ovary weight divided by the total weight times hundred. Ovary was kept for histological examination. The majority of individuals were in their intermolt, assessed by the lack of discernible gastroliths.

### Sample preparation and Illumina sequencing

Total RNA was extracted using Trizol Reagent (Invitrogen) following the manufacturer’s protocol. All RNA samples were checked on Bioanalyzer 2100 before sending for sequencing. Sequencing using HiSeq 2000 was conducted at BGI, Hong Kong as per manufacturer’s protocol (Illumina). Briefly, mRNA was isolated using oligo (dT) beads. All the mRNA resulted from fragmentation process were reverse transcribed into first strand cDNA using reverse transcriptase and random primers. Second strand of cDNA were then synthesized using DNA polymerase I and treated with RNAse H. All products were further purified and amplified using PCR to generate the final libraries. All prepared libraries were then sequenced using the Illumina HiSeq 2000, resulting with the final 90 bp paired-end libraries used in the current study. The data was stored in the form of FastQ files.

### Bioinformatics analyses

Seperately produced FastQ files from males and females were merged together to create one final raw file that represents the sequenced eyestalk transcriptome. FastQ qualities were assessed using FastQC software. (http://www.bioinformatics.babraham.ac.uk/projects/fastqc/). For maximum discovery of transcripts, the current study consisted of two different *de novo* assembly approaches. The first approach deployed a cleaning of low quality reads using Trimmomatic^42^ with the command parameter “*LEADING* = *3, TRAILING* *=* *3, SLIDING WINDOW* *=* *4,15, MIN SIZE* *=* *30*, *HEADCROP* *=* *6*”. Followed by a *de novo* assembly using Trinity_r20140717^43^ with the parameter “*–seqType fq –left Cq_EY_1_QCed.fastq –right Cq_EY_2_QCed.fastq –CPU 32 –JM 250* *G –normalize_reads –min_kmer_cov 2 –min_contig_length 200 –bfly_opts –PasaFly –group_pairs_distance 700 –extended_lock*”. The second approach was done mostly using a pre-designed workflow available in the CLC Genomics Workbench v8.0.3 (https://www.qiagenbioinformatics.com/). Briefly, raw reads were filtered based on PHRED score (>30), then loaded onto the CLC *de novo* assembly module, running with parameters: word size = 25, bubbles size = 50, minimum contig length = 200 bp. The final reference transcriptomes were then blasted against the NCBI non-redundant (nr) database for annotation of transcripts using BLAST+^44^. For searching of neuropeptides in *C. quadricarinatus*, all annotated sequences were scanned for keywords of previously known neuropeptides for instance “allatostatin”, “neuroparsin”, “corazonin”, or a known conserved amino acid motif such as “PSGFLGR”; list of motif used was taken as described elsewhere^10^. These sequences were then re-validated using blastp algorithm. For illustration purposes, cDNA sequences were converted to amino acids using Expasy translate tool available online (http://web.expasy.org/translate/), open reading frames (ORFs) were then chosen and loaded onto a pre-designed pipeline employing SignalP 4.0^45^ to validate they have a signal peptide and TargetP 1.1^46^ to confirm the neuropeptide is in the secretory pathway. Amino acid sequences of neuropeptides were saved as Fasta files for ease of analysis (Supplementary file 1). Amino acid sequences were submitted to the NeuroPred program^47^ to predict peptide cleavage sites. Schematic diagrams of neuropeptide structures were illustrated using the Illustrator for Biological Sequences (IBS) suite^48^. Multiple sequence alignments were done using CLC Genomics Workbench v8.0.3 (https://www.qiagenbioinformatics.com/). Multiple alignment files were also imported into MEGA 7.0^49^ for phylogenetic analysis. A maximum likelihood based approach was conducted with 500 bootstrap trials. Genbank accession number of all used sequences can be found in Supplementary file 2.

### Tissue distribution using RT-PCR

RT-PCR was performed as previously reported with slight modifications^50^. Out of the 10 individuals sampled above, RNA from different tissues of one mature animal with developed ovary (intermolt stage, weight 52.4 g, GSI = 4.2, oocyte diameter = 2.73 mm) was used to synthesize the complementary DNA libraries using Trizol Reagent (Invitrogen), following the manufacturer’s protocol. In total, 7 tissues including: eyestalk, brain and thoracic ganglia (combined), ovary, heart, abdomen muscle and antennal gland were used for PCR amplification to establish tissue distribution, using 14 neuropeptide gene primers. These neuropeptides were chosen based on literature review highlighting them as candidates for reproduction regulation in other crustacean species. Total RNA was re-extracted using the previously described method followed by cDNA synthesis using Tetro cDNA synthesis kit (Bioline, UK; using approximately 1 μg RNA per tissue). Amplifications were carried out using a touch-down PCR to allow most PCR products to be amplified with minimal non-specific signal. PCR settings were 94 °C for 3 min, followed by 37 cycles of touch down, 94 °C for 30 s, 62–57 °C for 30 s (with 1 °C decrement for each of the first 6 cycles) and 72 °C for 45 s. *Cherax quadricarinatus* Beta-actin (GenBank Accession number AY430093.1) was chosen as the house-keeping gene for this experiment. Primers used for all neuropeptides are listed in Supplementary file 3. Following PCR, products were loaded onto an agarose gel with ethidium bromide (1.5% TBE, EtBr concentration 10 mg/ml), electrophoresed for 30 min in 120 V, 0.4 mA and later visualized under UV-light.

## Results

### Illumina sequencing and *de novo* assembly

In total, the *C. quadricarinatus* pooled males and female libraries resulted in more than 200 million reads with the designated read length of 90 bp (paired end). All raw reads were submitted to the NCBI SRA under Accession: SRP091408. Read quality statistics including distribution of PHRED score, read length distribution, consistent GC content between samples and N bases in each tissue library were performed (Supplementary file 4). After trimming, the pooled sequenced library had more than 90% reads above the quality threshold (data not shown). Only quality-trimmed reads were then used in all downstream analyses.

In order to maximize the potential to discover neuropeptide transcripts, two different *de novo* assemblers were used. *De novo* assembled transcriptome of the current dataset using either Trinity or CLC *de novo* assembler yielded a large amount of transcripts. A brief summary of the *de novo* assembly statistics is provided in Table 1. All reads were subjected to the BLAST+ program and scanned against the NCBI nr database for hits. Neuropeptide transcripts were then manually chosen based on a number of criteria including N-terminal signal peptide availability, presence of previously known motifs and completeness of transcripts.

### Neuropeptide discovery by transcriptome mining: bioinformatics analysis and peptide prediction

Using a number of available bioinformatics packages, we were able to identify 53 predicted neuropeptide transcripts from the eyestalk of *C. quadricarinatus*, including most of the neuropeptides that were previously identified in other crustacean/insect species (Table 2). A comparative catalogue of neuropeptide sequences generated in the current study and similar characterization of neuropeptides from other crustacean species is also available in Supplementary material 5.

#### Adipokinetic hormone/Corazonin related peptide (ACP), allatostatin A, B (AST-A, B), allatostatin C/Prohormone 1 and allatostatin CC (AST-C, AST-CC), bursicon-α (partial) and CCAP

**Figure 1:**
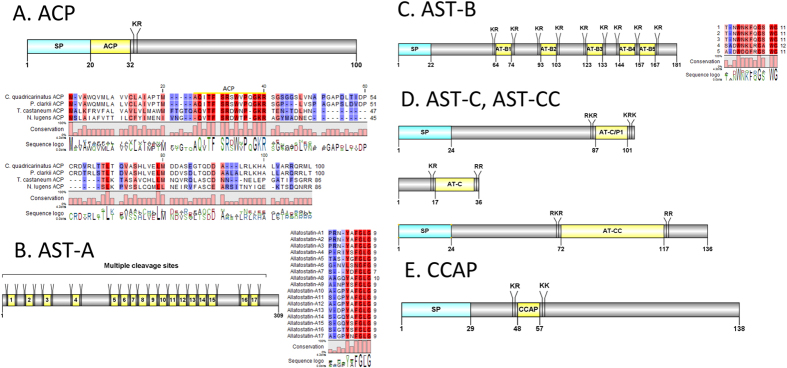
Molecular characterization of *Cherax quadricarinatus* ACP (**A**), allatostatin-A (**B**), allatostatin-B (**C**), allatostatin-C/allatostatin-CC (**D**) and CCAP (**E**). Schematic diagrams show structure of neuropeptide precursors, including signal peptide (SP), the mature peptide (yellow) and putative cleavage sites. Precursor sequence alignments are shown with site of the mature peptide highlighted in yellow. Conserved amino acids are shown in a gradient from blue to red (blue means nearly similar, dark red means exact amino acid and white is no conservation).

A putative ACP precursor can be deduced from the *de novo* transcriptome assembly of *C. quadricarinatus* eyestalk (Fig. 1A). The predicted neuropeptide is 12aa long, immediately following a 20aa signal peptide and preceding a dibasic cleavage K_33_R. Global alignment of *Cq*-ACP with ACP from other related species shows most significant conservation within the ACP mature peptide. We detected a partial transcript (without signal peptide) that encoded for the precursor of *Cq*-AST-A (Fig. 1B), containing 17 predicted peptides flanked by multiple dibasic cleavage sites. The conserved motif recorded was X**Y**X**FLGamide**, which is similar to insect and other crustacean AST-A. One *Cq*-AST-B transcript was found in our *de novo* assembly (Fig. 1C). The neuropeptide precursor has a 22aa signal peptide and six dibasic cleavage sites that, if processed, are predicted to release 5 mature peptides of 11–12aa, and contain the conserved X**W**XXXX**G**X**Wamide**motif, also observed in insects and other crustaceans^51^. Three isoforms of *Cq-AST-C* were found in the eyestalk *de novo* assembly (Fig. 1D). First, an AST-C-like/Prohormone-1 precursor, with a 24aa signal peptide and a 15aa mature peptide, second, a 36aa *Cq*-AST-C was found without a signal peptide, with the conserved **PISCFamide**, and, finally a *Cq*-AST-CC was found with a predicted precursor of 138aa and a 46aa mature peptide. One transcript encoding a *Cq*-buriscon-α was identified in this study, although with no apparent signal peptide or cleavage site following the mature peptide, it is a partial sequence. The deduced amino acid sequence for Cq-buriscon-α detected from the *de novo* assembly is SGIFLSCPGQILTRAPIDCMCRPCTDVEEGTVLAQEIANFIHDSPMGNVPFLK. A 138aa *Cq*-CCAP precursor was deduced, containing a signal peptide and two cleavage sites (K_46_R and K_58_K) that could release a mature peptide **PFCNAFTGCamide** (Fig. 1E).

#### CCHamide (CCH), crustacean female sex hormone-like (CFSH-like), DH31, corazonin, DH44, eclosion hormone, elevenin

**Figure 2:**
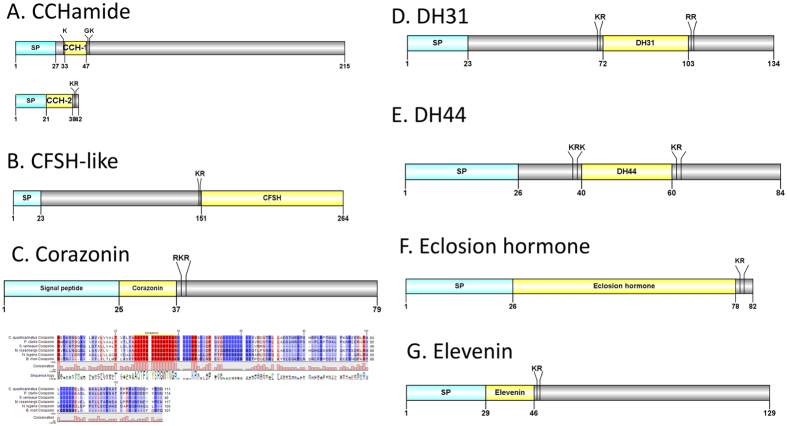
Molecular characterization of *Cherax quadricarinatus* CCHamide (**A**), CFSH-like (**B**), corazonin (**C**), DH31 (**D**), DH44 (**E**), eclosion hormone (**F**) and elevenin (**G**). Schematic diagrams show structure of neuropeptide precursors, including signal peptide (SP), the mature peptide (yellow) and putative cleavage sites. Precursor sequence alignments are shown with site of the mature peptide highlighted in yellow. Conserved amino acids are shown in a gradient from blue to red (blue means not similar, dark red means exact amino acid).

We identified two *Cq*-CCH transcripts in the current *de novo* transcriptome assembly of *C. quadricarinatus* (Fig. 2A). The two transcripts encode for 215aa and 42aa proteins, respectively, each with a signal peptide. *Cq*-CCH-1 has two cleavage sites (K_32_ and G_48_K), that if processed would release the mature peptide SCSQFGHSCFGAHamide, while the predicted *Cq*-CCH-2 mature peptide contains GGCLNYGHSCLGAHamide, which has a C-terminal K_39_R cleavage site. The conserved motif for *Cq-*CCHamide is **GHSC**X**GAHamide.** A putative CFSH-like transcript was detected in the eyestalk of *C. quadricarinatus*. *Cq*-CFSH-like precursor is 264aa in length, with a signal peptide and a K_149_R cleavage site, followed immediately with the mature peptide (Fig. 2B). A predicted corazonin precursor (*Cq*-Crz) includes a 25aa signal peptide, followed immediately by the *Cq*-Crz mature peptide **pQTFQYSRGWTNamide** and a R_37_KR cleavage site. Multiple sequence alignments show high similarity of *Cq*-Crz mature peptide with Crz of *M. rosenbergii*, *P. clarkii*, *S. verreauxi* and insects including *Bombyx mori* and *Nilaparvata lugens* (Fig. 2C). A *Cq*-DH31 precursor was deduced to be containing a precursor 23aa signal peptide, a dibasic K_70_R and R_104_R cleavage site, that if processed would release a 31aa mature DH31 peptide (Fig. 2D). A DH44 transcript encodes a precursor of 84aa, with a 23aa signal peptide and 31aa DH44 mature peptide processed from two dibasic cleavage sites (R_38_K and K_62_R) (Fig. 2E). A full-length transcript of eclosion prohormone can be deduced to be consisting of 82aa, including a 26aa signal peptide and a single mature peptide flanked C-terminally by a cleavage site (K_79_R) (Fig. 2F). A predicted elevenin precursor was identified in our study at 129aa in length, with a 29aa signal peptide and a 17aa mature peptide VDCRKFVFAPVCRGIIA (Fig. 2G).

#### Crustacean Hyperglycemic Hormone (CHHs) families

**Figure 3:**
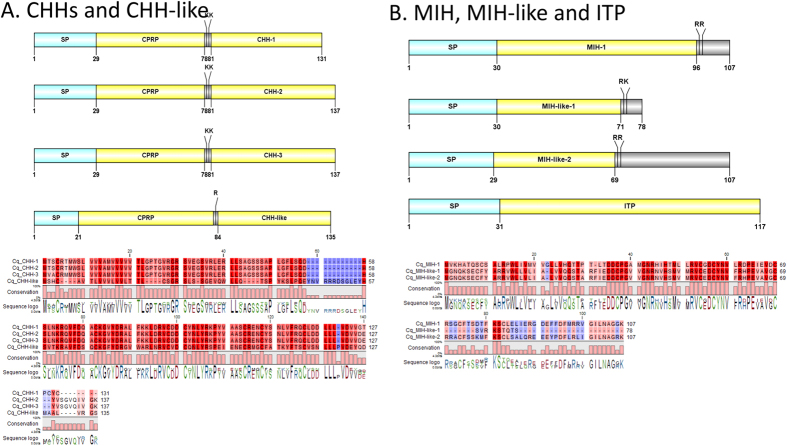
Molecular characterization of *Cherax quadricarinatus* CHHs and CHH-like (**A**), MIH, MIH-like and ITP (**B**). Schematic diagrams show structure of neuropeptide precursors, including signal peptide (SP), the mature peptide (yellow) and putative cleavage sites. Precursor sequence alignments are shown with site of the mature peptide highlighted in yellow. Conserved amino acids are shown in a gradient from blue to red (blue means nearly similar, dark red means exact amino acid and white is no conservation).

**Figure 4:**
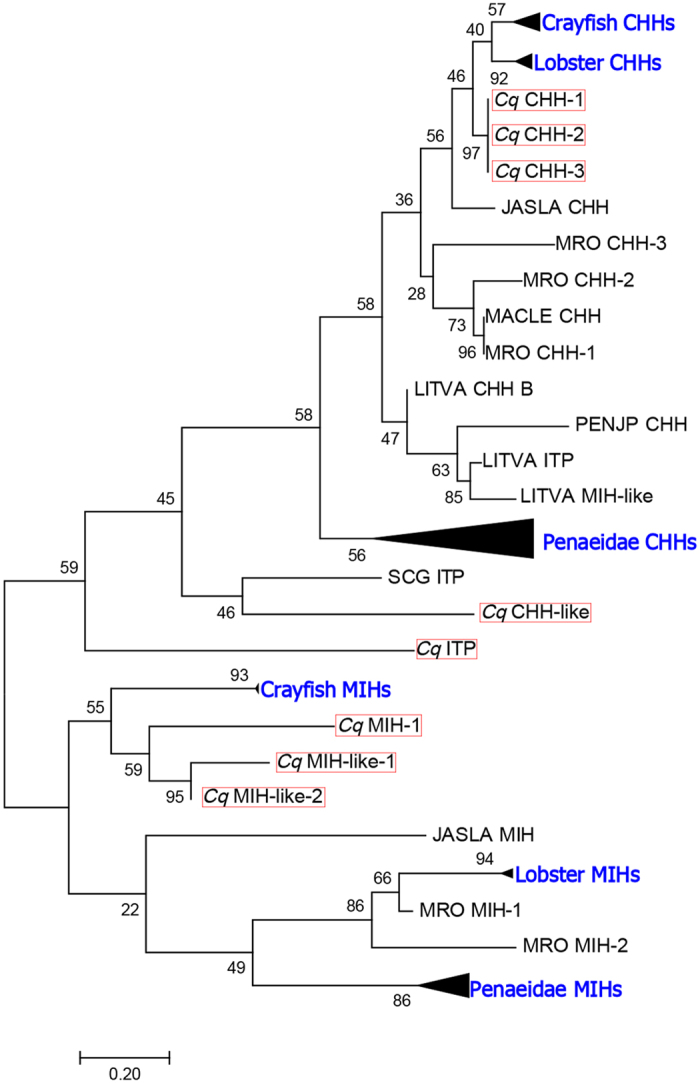
A maximum likelihood method based on the JTT matrix-based model was used to produce the phylogenetic tree using amino acids sequence of CHHs, MIHs and ITPs. 500 bootstrap replicates was used to produce the phylogenetic tree using amino acids sequence of CHHs/MIHs and ITP isoforms (that include signal peptide and the mature sequence). The tree is drawn to scale, with branch lengths measured in the number of substitutions per site. Bootstrap values (1–100) are given at each branch. *C. quadricarinatus* CHHs, MIH and ITP are highlighted in red boxes. JASLA: *Jasus lallandi*, LITVA: *Lipopanaeus vannamei*, PENJP: *Paneaus vannamei*, SCG: *Schistocerca gregaria*, MRO: *Macrobrachium rosenbergii*.

In total, three CHH isoforms and a CHH-like transcript were deduced from the eyestalk *C. quadricarinatus de novo* assembly (Fig. 3A). *Cq*-CHH-1, *Cq*-CHH-2 and *Cq*-CHH-3 all consist of a 29aa signal peptide, a CHH precursor-related peptide (CPRP) and a CHH mature peptide of 51aa, 57aa and 57aa, respectively. Moreover, one CHH-like peptide was also detected as containing a CPRP peptide and a CHH mature peptide that deviates in its composition from the other 3 CHHs. One *Cq*-MIH transcript and two *Cq*-MIH-like transcripts were identified (Fig. 3B); *Cq*-MIH-1 is identical to a previously described *Cq*-MIH (Accession ACX55057). The two *Cq*-MIH-like transcripts are 78 and 107aa in length, each with a signal peptide and cleavage sites predicted to release a 42aa and 41aa mature peptide, respectively. Among the CHH families, we identified one putative *Cq*-ITP that has a 31aa signal peptide, and a single mature peptide of 87aa. A phylogenetic tree of the *Cq*-CHH superfamily and CHH peptides from related species was constructed (Fig. 4). Based on this tree, *Cq*-CHH is allocated within the same cluster as lobster CHHs (including *Homarus gamarus*, *Nephrops norvergicus*, *Jasus lallandi*), while CHHs from other crayfish species, including *Procambarus* spp., are distributed into another clade. In contrast, *Cq*-MIH groups with crayfish MIHs and is quite distinct from lobster MIHs. The two *Cq*-CHH-like and *Cq*-ITP cluster together as a middle clade, suggesting they are a hybrid form between CHH and MIH.

#### FLRFamide, GPA2/GBP5, GSEFLamide, HIGSLYamide, insulin, kinin and myosuppressin

**Figure 5:**
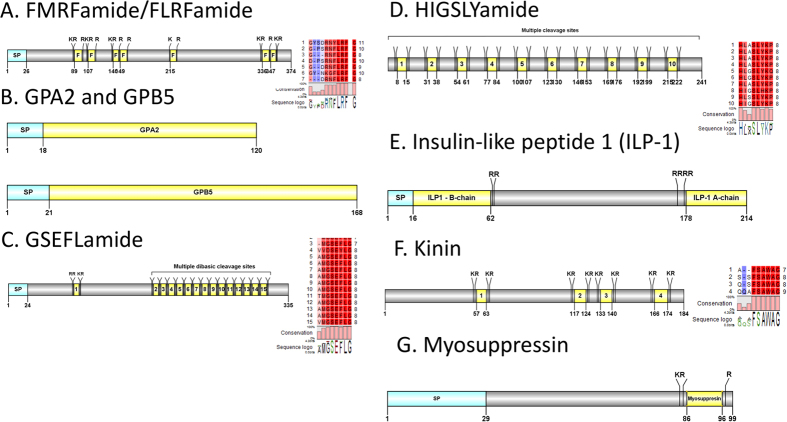
Molecular characterization of *Cherax quadricarinatus* FLRFamide (**A**), GPA2 and GPB5 (**B**), GSEFLamide (**C**), HIGSLYamide (**D**), Insulin-like peptide (**E**), Kinin (**F**), Myosuppressin (**G**). Schematic diagrams show structure of neuropeptide precursors, including signal peptide (SP), the mature peptide (yellow) and putative cleavage sites. Precursor sequence alignments are shown with site of the mature peptide highlighted in yellow. Conserved amino acids are shown in a gradient from blue to red (blue means nearly similar, dark red means exact amino acid and white is no conservation).

A predicted *Cq*-FLRFamide precursor consists of a 26aa signal peptide, and processing sites that could release up to 7 mature peptides ranging in size from 8–11aa (Fig. 5A). The FLRFamide motif in *C. quadricarinatus* is **X-Y/F-LRFamide.** We report a putative GPA2/GBP5 (Fig. 5B). The deduced GPA2 is 120aa in length, with an 18aa signal peptide, followed immediately by a 103aa mature peptide. The *Cq*-GBP5 is larger, at 168aa, and consists of a 21aa signal peptide and 147aa GPB5 mature peptide. A *Cq*-GSELFamide transcript encodes for a 335aa precursor, containing a 24aa signal peptide and followed by 15 mature peptides flanked by multiple cleavage sites (Fig. 5C). The deduced mature peptides vary in length from 7–8aa and each have a conserved X**GSEFLamide**motif. A partial precursor sequence for HIGSLYRamide was identified, consisting of 241aa and no signal peptide (Fig. 5D). Multiple dibasic KR/RR cleavage sites suggest the presence of 10 mature peptides, each 8aa in length. The conserved motif within each peptide is **H**L/IA/**GSL**Y/H**Kamide** (Fig. 5D). A complete identical Insulin-like peptide previously characterised in *C. quadricarinatus* was reconfirmed (Accession AIU40992). The neuropeptide is 214aa in length, contains a signal peptide and 2 conserved regions that would be the mature hormone (Fig. 5E). One partial kinin precursor was identified in our *de novo* assembly (lacking the signal peptide) (Fig. 5F). The precursor is 184aa in length and has 4 predicted mature peptides, separated by multiple dibasic cleavage sites. The conserved motif detected is X**FSAWAamide**. A myosuppressin precursor was deduced from the *de novo* assembly (Fig. 5G). The precursor consists of a 29aa signal peptide, a K_84_R and R_97_ cleavage site that resulted in an 11aa mature myosuppressin (QDLDHVFLRFamide). *Cq*-myosuppressin has the same motif found in other known crustacean species.

#### Neuroparsin, Neuropeptide F, Orcokinin, pigment dispersing hormone (PDH), pyrokinin, prohormone-4, proctolin

**Figure 6:**
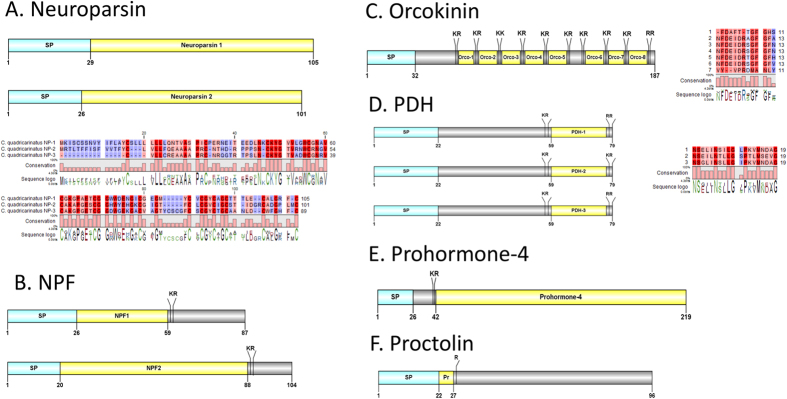
Molecular characterization of *Cherax quadricarinatus* Neuroparsin (**A**), NPF (**B**), PDHs (**C**), FRLFamide (**D**), Prohormone-4 (**E**) and Proctolin (**F**). Schematic diagrams show structure of neuropeptide precursors, including signal peptide (SP), the mature peptide (yellow) and putative cleavage sites. Precursor sequence alignments are shown with site of the mature peptide highlighted in yellow. Conserved amino acids are shown in a gradient from blue to red (blue means nearly similar, dark red means exact amino acid and white is no conservation).

**Figure 7:**
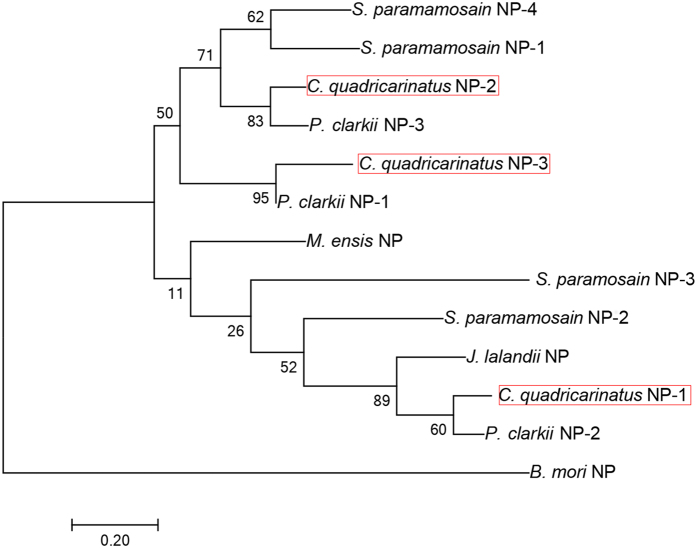
Molecular phylogenetic analysis of Neuroparsin isoforms by Maximum Likelihood method based on the JTT matrix-based model. 500 bootstrap replicates was used to produce the phylogenetic tree using amino acids sequence of prepro-neuroparsin (that include signal peptide and the mature sequence) The tree is drawn to scale, with branch lengths measured in the number of substitutions per site. Bootstrap values (1–100) are given at each branch. *C. quadricarinatus* Neuroparsins are highlighted in red boxes.

Three *Cq*-NP transcripts were found (Fig. 6A), including two full-length sequences with signal peptides of 29aa and 26aa. The predicted mature peptides of 75aa and 80aa are encoded immediately after the signal peptide. A phylogenetic tree analysis of NP precursors was constructed, showing that *Cq*-NP2 clusters strongly with *P. clarkii*-NP3 (Pc-NP3), alongside *S. paramamosain-*NP1 (*Sp*-Np1) and *Sp*-NP4 (Fig. 7). Less well clustered, yet within the same branch, *Cq*-NP3 (partial-length) clusters with *Pc*-NP1. Together these form a clade distinct from *Cq*-NP1, which clusters strongly with *Pc*-NP2, and also cluster with *Sp*-NP2 and *Sp*-NP3. For this tree, the silk moth *Bombyx mori* NP served as an outgroup. Two sequences of *Cq*-NPF were identified in our *de novo* assembly with 87 and 104aa, including a signal peptide of 26 and 20aa, respectively (Fig. 6B). The predicted mature peptide starts immediately right after the signal peptide and both of them have the conserve motif **RPRFamide.** We identified one orcokinin precursor 187aa. Orcokinin has a predicted signal peptide of 32aa and multiple cleavage sites that potentially releases up to 8 mature peptides. Of those, there exists a conserved C-terminus motif X**FDEIDR**X**GFGF**X**amide** (Fig. 6C), which is similar to other known crustacean species orcokinins - NFDEIDRSGFGFNamide^10^. Three PDH precursors were deduced from the eyestalk transcriptome, namely *Cq*-PDH-1, *Cq*-PDH-2 and *Cq*-PDH-3 (Fig. 6D). All have a 22aa signal peptide, followed by a dibasic K_57_R and R/K_78_R cleavage site that could release a mature peptide of 18aa. A prohormone-4 transcript was detected in our study, encoding a 219aa precursor that contains a 26aa signal peptide and K_40_R cleavage site that could release a 178aa mature peptide (Fig. 6E). A proctolin transcript was identified that encodes a 96aa precursor, encoding a 22aa signal peptide followed immediately by the *Cq*-Proctolin mature peptide (Fig. 6F). *Cq*-Proctolin has a **RYLPT** motif, similar to other crustacean species^10^.

#### Pyrokinin, RYamide, SIFamide, short neuropeptide F, sulfakinin (SK), tachykinin, WXXXRamide

**Figure 8:**
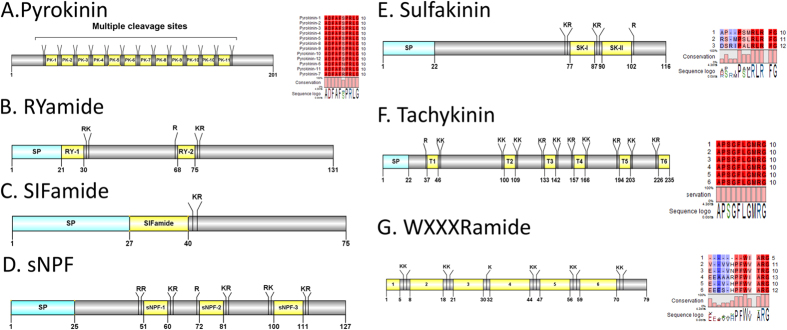
Molecular characterization of *Cherax quadricarinatus* Pyrokinin (**A**), Ryamide (**B**), SIFamide (**C**), sNPF (**D**), Sulfakinin (**E**), Tachykinin (**F**) and WXXXRamide (**G**). Schematic diagrams show structure of neuropeptide precursors, including signal peptide (SP), the mature peptide (yellow) and putative cleavage sites. Precursor sequence alignments are shown with site of the mature peptide highlighted in yellow. Conserved amino acids are shown in a gradient from blue to red (blue means nearly similar, dark red means exact amino acid and white is no conservation).

A partial *Cq*-pyrokinin precursor that lacks a signal peptide, yet contains multiple dibasic cleavage sites that can release 11 highly conserved mature peptides – **ADFAF**X**PRLamide,** was deduced from the eyestalk transcriptome (Fig. 8A). The 5aa C-terminal region **F**X**PRLamide** is highly conserved among the pyrokinin/pheromone biosynthesis activating neuropeptide (PBAN) family^52^. A transcript encoding for a 131aa *Cq*-RYamide precursor was deduced from our data (Fig. 8B). The precursor contains a 21aa signal peptide, followed by two mature peptides, QGFYSQRYamide and FIGGSRYamide, which share a conserved XXXXX**RYamide**motif. One transcript encoding for *Cq*-SIFamide was identified. The preprohormone start has a signal peptide of 27aa, followed by the mature peptide with a C-terminal dibasic cleavage site K_41_R (Fig. 8C). The mature peptide, GYRKPPFNGSIFamide, shares significant sequence similarity with other known SIFamides: GYRKPPFNGSIFamide^10^. One transcript was identified that encodes a 127aa sNPF precursor, containing a 25aa signal peptide, followed by three dibasic cleavage sites that could release three mature sNPF peptides (Fig. 8D). The mature peptides share the conserved motif X**P**X**RLRFamide**. The 116aa *Cq*-sulfakinin is composed of a 22aa signal peptide and two sulfakinin mature peptides GGDYDDYGHLRRFamide and EFDEYGHMRFamide, which share a common C-terminal **DYGH**X**RFamide** (Fig. 8F). A *Cq*-Tachykinin (TK) transcript was identified, encoding a precursor with a 22aa signal peptide, followed by 6 TK peptides (9aa), each flanked by cleavage sites (Fig. 8G). The predicted TK peptides all share the conserved **APSGFLGMRamide**motif, a feature consistent with other crustacean species TK peptides. A partial transcript of *Cq*-WXXXRamide was detected in our eyestalk *de novo* transcriptome, which encodes a precursor with no signal peptide and multiple cleavage sites that could release up to 6 mature peptides with the C-terminus **W**XXX**Ramide**motif.

#### Red pigment concentrating hormone (RPCH), vasopressin-neurophysin

**Figure 9:**
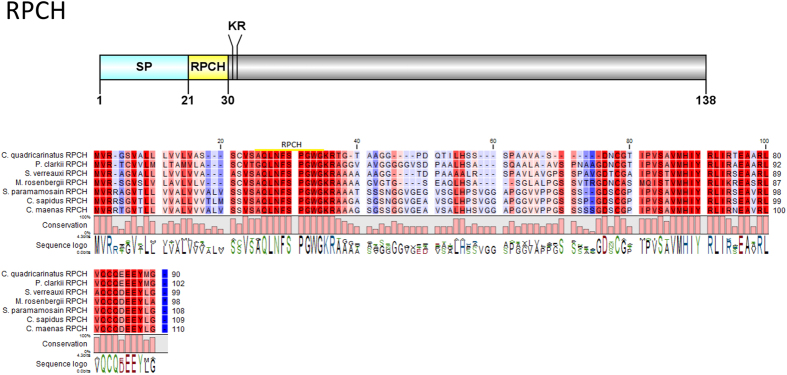
Molecular characterization of *Cherax quadricarinatus* RPCH. Schematic diagrams show structure of neuropeptide precursors, including signal peptide (SP), the mature peptide (yellow) and putative cleavage sites. Precursor sequence alignments are shown with site of the mature peptide highlighted in yellow. Conserved amino acids are shown in a gradient from blue to red (blue means nearly similar, dark red means exact amino acid and white is no conservation).

**Figure 10:**
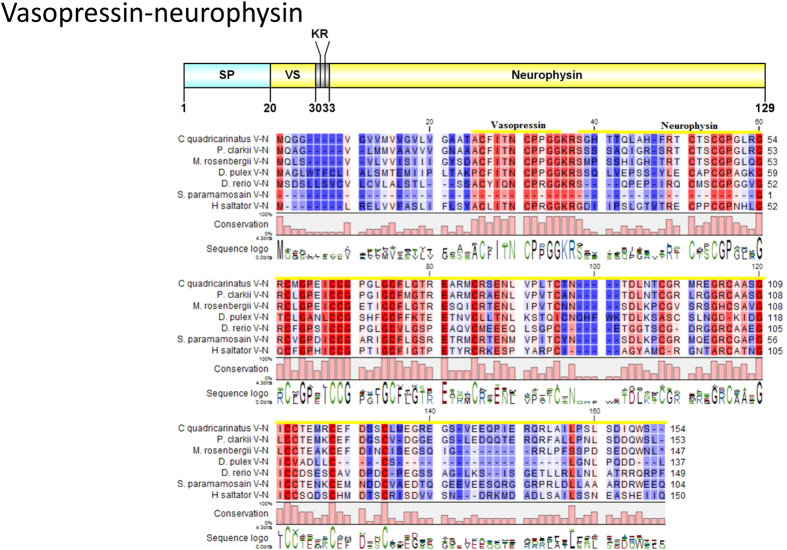
Molecular characterization of *Cherax quadricarinatus* Vasopressin-Neurophysin. Schematic diagrams show structure of neuropeptide precursors, including signal peptide (SP), the mature peptide (yellow) and putative cleavage sites. Precursor sequence alignments are shown with site of the mature peptide highlighted in yellow. Conserved amino acids are shown in a gradient from blue to red (blue means nearly similar, dark red means exact amino acid and white is no conservation).

A single *Cq-RPCH* transcript was deduced from the eyestalk transcriptome (Fig. 9). The RPCH precursor consists of a 21aa signal peptide, followed immediately by a 9aa mature peptide **QLNFSPGWamide** and a dibasic cleavage site at K_31_R. The predicted mature peptide is very similar to known RPCH in other crustacean species (Fig. 9). A vasopressin-neurophysin preprohormone containing a 20aa signal peptide, followed by a vasopressin peptide and a neurophysin mature peptide was also identified (Fig. 10). Multiple sequence alignment shows significant identity of *Cq*-vasopressin-neurophysin with other closely related insect and crustacean species.

#### GnRH superfamily

**Figure 11:**
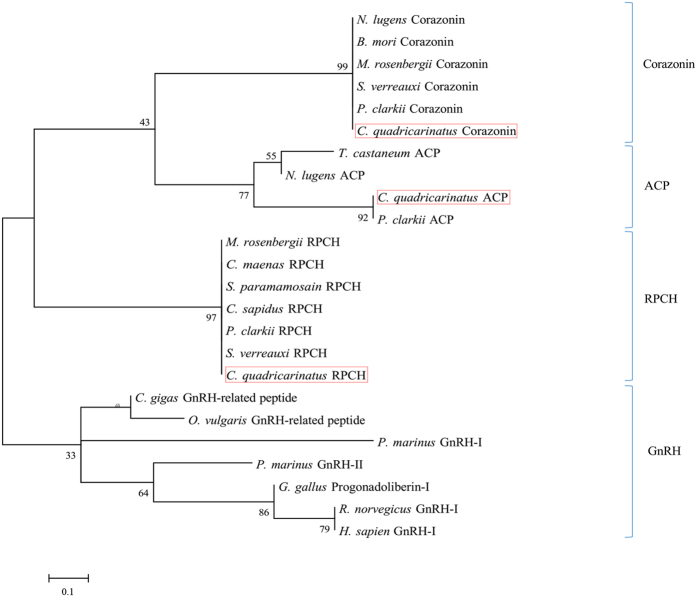
Molecular phylogenetic analysis of GnRH superfamily by Maximum Likelihood method based on the JTT matrix-based model. 500 bootstrap replicates was used to produce the phylogenetic tree using mature amino acids sequence of RPCH, Corazonin, ACP and GnRH. *C. quadricarinatus* neuropeptide are highlighted with red boxes.

In our study, we used the mature peptide sequence of *Cq*-ACP, *Cq*-RPCH, *Cq*-Crz, and other related species ACP, RPCH, Crz as well as GnRH to construct a phylogenetic tree for the GnRH superfamily (Fig. 11). The tree branched into 4 distinct groups ACP, RPCH, Crz, GnRH/GnRH-like (highlighted in red). The analysis indicated that RPCH, ACP and Crz mature peptides are more closely related to each other compared with GnRH, located within the same clade, while GnRHs are well clustered within their own clade.

### Tissue distribution using RT-PCR

**Figure 12:**
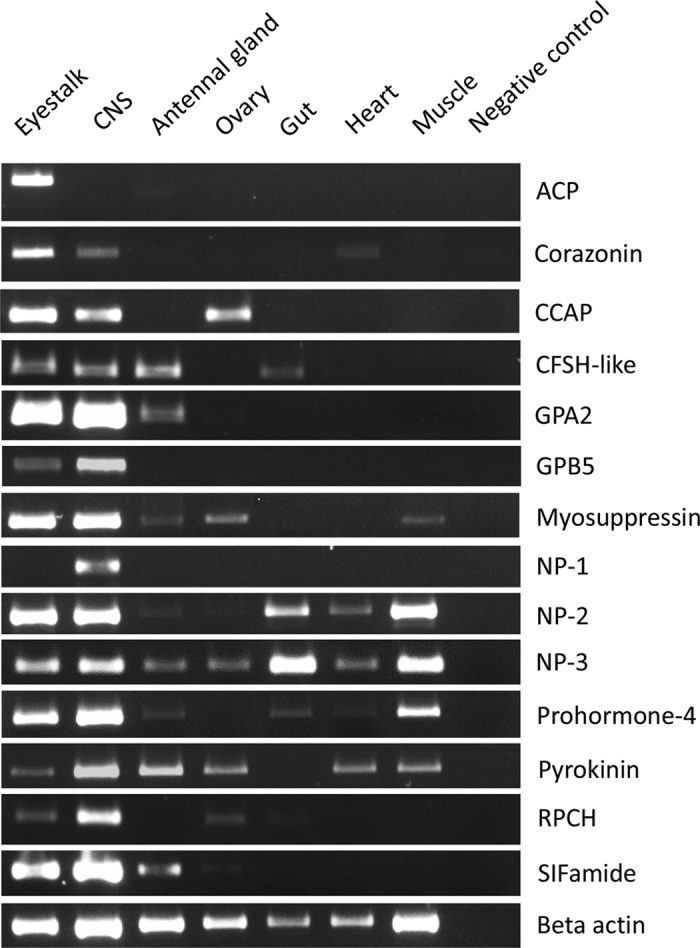
Tissue specific expression of *C. quadricarinatus* neuropeptide genes using RT-PCR. Expression of 14 neuropeptide genes using gene-specific primers, as well as Beta-actin gene. PCR used cDNA derived from tissues of a mature female. Negative control represents no cDNA in PCR. CNS: Central nervous system (Brain + Thoracic ganglia), ACP: Adipokinetic/Corazonin-retated peptide, CCAP: Crustacean cardioactive peptide, CFSH-like: Crustacean female sex hormone-like, GPA2: Glycoprotein alpha-2, GPB5: Glycoprotein beta-5, NP: Neuroparsin, RPCH: Red pigment concentrating hormone, sNPF: Short neuropeptide F. All the gels were run under the same experimental conditions and are presented using cropped images. The entire gel photos of all neuropeptide genes can be find in Supplementary material 6.

An RT-PCR experiment was designed to map the expression of 14 of the neuropeptide genes in 7 different *C. quadricarinatus* tissues, including supraesophageal ganglia (referred to as the brain) and thoracic ganglia (combined and referred to as the central nervous system; CNS), eyestalk, heart, gut, antennal gland, ovary and muscle (Fig. 12). Most neuropeptide amplicons were detected in all the neural-type tissues (eyestalk, brain and thoracic ganglia), except for the *NP-1* isoform that was expressed in the CNS exclusively. Also, the *ACP* gene was expressed in CNS, and with some expression observed in the antennal gland. Seven of the neuropeptide genes tested were found to be expressed in the ovary, including *CCAP, myosuppressin, NP-2, NP-3, pyrokinin, RPCH,* and *SIFamide*. As expected, beta-actin showed up in all tissues tested except negative control.

## Discussion

This study presents an RNA sequencing approach to characterise a catalogue of neuropeptide genes in the eyestalk of the Australian red claw crayfish *C. quadricarinatus*. To identify the neuropeptides, two different *de novo* assemblers were employed to maximize transcript discovery as well as completeness. It is now well established that no single assembler is superior in assembly quality when compared with the others, as different assemblers provide different algorithms to address bubble effects, mismatches, and errors, to name a few, that each require different options and optimized parameters^53^,^54^,^55^. We observed that by having two *de novo* assemblies instead of one, we had greater success in obtaining full-length transcripts. Even with 2 assemblies, for some neuropeptides (e.g. Bursicon, HIGSLYRamide, Kinin, Allatostatin-C), we still could only detect partial-length sequences, possibly due to the fact that crustacean eyestalks are rich in secondary metabolites including phenolic compounds^56^, which can compromise RNA quality.

In the absence of a genome sequence, data mining using transcriptomics data is a powerful tool for neuropeptide discovery in crustacean species^5^,^6^,^7^,^8^,^9^. Our *in silico* data mining of both eyestalk assemblies resulted in the identification of 53 neuropeptide-encoding transcripts, of which 14 were further assessed for tissue expression using RT-PCR. A number of neuropeptides genes detected in the current study are very similar to those identified in other *in silico* studies that have investigated related crustacean or insect species. Confirmation of neuropeptide genes in a diverse range of species is important since several arthropod neuropeptides have an unknown function, while others have a function that has only been partially confirmed or appear to have ambiguous roles^27^,^28^,^29^,^30^. A similar recent *in silico* analysis approach in *M. rosenbergii* central nervous system yielded 21 neuropeptide transcripts (with some having multiple isoforms) that encode up to 102 mature peptides^6^. Another transcriptome-based neuropeptide study on *P. clarkii* mined 58 different neuropeptide transcripts, as well as their putative receptors^7^. Analysis of the central nervous system transcriptome in *S. verreauxi* revealed 37 transcripts representing 21 peptide/protein subfamilies^9^. In *H. americanus*, 35 precursors that released 194 distinct neuropeptides were detected from the neural tissues^8^. A very recent comprehensive collection of neuropeptides in a number of decapod species showcase a comprehensive list of characterised transcripts^57^ with high similarity between species, concluding that the gap between insects and crustaceans is narrow as far as the neuropeptide repertoire is concerned. A number of neuropeptides described in the above study by Veenstra were not detected in our dataset including Calcitonins, CFSH, CCRFamide, DILP7-like, Periviscerokinin and Trissin. In the current study, we mined a large number of neuropeptides, (comparison can be found in Supplementary Material 5), however it is important to note that in the absence of a sequenced genome, the number of neuropeptides mined, as well as sequencing completeness can be elusive, since it is dependent on the timing of expression and tissues chosen – which in our case, is solely based on the eyestalk ganglia, but not the entire CNS of the species.

In the current study, we highlighted a number of neuropeptides that have predicted roles in reproduction and sexual development. In our study, we were able to identify the mature peptide regions for *Cq*-AST-A, B, C, which opens up avenues for functional analysis in *C. quadricarinatus*. AST has a pleiotropic role in insects, for which one function is inhibition of JH synthesis by the corpora allata^51^. A precursor named methyl farnesoate (MF) is considered the crustacean equivalent of the insect JH. However, recent findings of the metabolizing enzyme which converts MF to JH – CYP15A1 – in *M. rosenbergii*^58^ and *S. verreauxi*^59^, specifically in the antennal gland (which is linked to the mandibular organ, the production site of MF), supports the notion that JH is an active hormone is crustaceans as well. Additionally, *Diploptera punctata* AST showed a stimulatory effect on MF synthesis in the mandibular organ of the adult crayfish *P. clarkii*^60^. Silencing AST-A reduced egg and testes development in crickets, and the oviposition rate was drastically diminished in both species^61^. AST-C may be of particular interest since it has been proven previously to be a key factor in JH synthesis inhibition in different insects^62^,^63^. Interestingly, a recent study has shown that local gene duplications in an ancestral arthropod created three allatostatin C genes, namely allatostatin-C, CC and CCC, with all three genes present in decapods^64^. The pattern is similar to our results, where we can predict 3 allatostatin-C isoforms that are distinct from each other. Further research is warrant to investigate differences in functionalities of these genes.

From our results, one bursicon-α partial transcript was deduced from the eyestalk *de novo* assemblies, while we could not retrieve any bursicon-β sequence. This can be elucidated by tissue expression at various physiological stages, since in both *M. rosenbergii* and *Carcinus maenas*, bursicon-β could only be found in the thoracic ganglion but not eyestalk^6^,^65^. Bursicon is a neuropeptide found throughout arthropods that was shown to regulate cuticle tanning, hardening and wing expansion of insects following ecdysis^66^. In contrast, much less information is available about bursicon function in crustaceans. However, it is known that injection of recombinant bursicon-β into female *P. monodon* broodstock causes an increase in vitellogenin gene expression and stimulated ovarian development^28^.

Crustacean cardioactive peptide (CCAP) is a neuropeptide that is highly conserved between crustacean species, with all containing the PFCNAFTGCamide motif. It has been demonstrated to have a wide range of functions in insects as well as crustaceans including cardiac control^67^, adaptation to environment stressors^68^, stimulation of the oviduct^69^, regulation of gut tissues^70^ and ecdysis^71^,^72^. In *S. paramamosain*, up-regulation of CCAP increased haemolymph circulation, and the authors suggested that CCAP facilitates movement of vitellogenin into the ovary during its reproduction phase^5^. In our study, we detected CCAP expression in the CNS, eyestalk and ovary of *C. quadricarinatus*, a similar fashion to that of *M. rosenbergii*^6^, further implicating CCAP role in reproduction.

Crustacean female sex hormone (CFSH) was recently confirmed to be involved in female sexual maturation in the blue crab *C. sapidus*^17^. In that study, it was demonstrated that CFSH was highly expressed in females but not males, and has a crucial role in developing the female phenotype^17^. In our *de novo* transcriptome, no match for CFSH was detected within the designated threshold. However, we were able to identify a transcript that is very similar to a CFSH-like neuropeptide previously described in *P. clarkii*^7^. That CFSH, whose structure is somewhat related to the mud crab female sex hormone, appears to be expressed exclusively in the eyestalks^7^. From RT-PCR results in our study, we have shown that *Cq*-CFSH-like is not eyestalk exclusive, but is also expressed in the CNS, antennal gland and gut, but not ovary, from one mature individual. Still, the specific role of the CFSH-like peptide in *C. quadricarinatus* and whether it contributes toward reproduction requires further investigation.

Crustacean hyperglycemic hormones (CHHs) are an important superfamily of peptides in crustaceans, produced in the X-organ, then transported to the sinus gland, where they are stored and subsequently secreted, together forming the eyestalk XO-SG complex. CHHs have been extensively studied, revealing diverse roles in carbohydrate metabolism, osmoregulation, reproduction and molting [for reviews, see Chan, *et al*.^13^, Christie, *et al*.^10^,^12^,^25^]. In crustaceans, multiple CHH isoforms have been detected in neuropeptidomes. For instance, studies have found 2 isoforms in *S. paramamosain*^5^, 3 isoforms in *M. rosenbergii*^6^ and *P. clarkii*^7^, and 4 isoforms in *H. americanus*^8^ and *S. verreauxi*^9^. Three CHH isoforms were deduced in this study, each with six conserved cysteine residues, which give rise to three intramolecular disulfide bridges^12^. Meanwhile, the CHH family member representative *Cq*-MIH is identical to the MIH previously described also in *C. quadricarinatus*^73^.

Myosuppressin belongs to a group of neuropeptides that are only found in insects or crustaceans. It has previously been demonstrated to exhibit several biological activities, including inhibition of gut contractions^74^, antifeeding activity^75^, and inhibition of neuropeptide secretion^76^ and has been suggested previously to be a brain/gut peptide^75^. In *C. quadricarinatus*, we detected expression of myosuppressin in the CNS, eyestalk, antennal gland, muscle and most surprisingly, the ovary in a mature female, showing for the first time a potential link between myosuppressin expression and reproduction.

Another group of neuropeptides which are associated with reproduction are neuroparsins (NPs), which have diverse functions in insects, including delayed vitellogenesis^77^. In crustaceans, a number of NP isoforms have been detected in *M. rosenbergii*^6^, *P. clarkii*^7^, *S. paramamosain*^5^ and several decapod species^57^. From the current study, three NP isoforms were deduced; *Cq*-NP-1 and *Cq*-NP-2 have 12 cysteine residues (possibly forming 6 disulfide bridges), while *Cq*-NP-3 is a partial sequence with 16 cysteines. Tissue screening using RT-PCR showed that *Cq-NP-2* and *Cq-NP-3* transcripts are widely distributed in most of the tissues we tested, while surprisingly, *Cq-NP-1* expression is exclusive to the CNS. We propose that this could be a stage-specific Neuroparsin, however further qPCR will be needed to elucidate the expression pattern of this specific neuropeptide. *In vivo* gene silencing of one NP isoform in the sand shrimp *Metapenaeus ensis* caused a significant decrease in hepatopancreas and ovary vitellogenin transcript levels^27^, supporting a reproduction related role while in *S. paramamosain*, gene expression profiling on Sp-NP1, Sp-NP2 and Sp-NP3 suggests that these neuropeptide have a stimulating effect on early stage vitellogenesis and Sp-NP4 in late stage vitellogenesis^5^.

A single *Cq-SIFamide* transcript was identified in the *C. quadricarinatus* eyestalk, CNS, antennal gland and ovary. Most recently, upregulation of SIFamide was detected during the early vitellogenic stages in the mud crab *S. paramamosain*^5^, while in the prawn *M. rosenbergii*, it has been suggested that SIFamide modulates aggression, in association with adult courtship behaviour^78^. SIFamide is a well conserved neuropeptide in insects and crustaceans^79^. In insects, SIFamide has been proven to have a role in sexual behaviour^80^ and promote sleep in *Drosophila melanogaster*^81^.

The PBAN/pyrokinin family is a major group of insect and crustacean neuropeptides that have been implicated in multiple functions during development, mating and reproduction^52^,^82^,^83^. Pyrokinin has now been found in several different species across insects to crustaceans. Our RT-PCR indicated that *Cq-pyrokinin* is present in most of the tissues screened (except gut), suggesting an important regulatory role of pyrokinin in a wide array of biological processes in *C. quadricarinatus*. An effort to silence pyrokinin in *Solenopsis invicta* and *Helicoverpa zea* showed negative impacts post-silencing, including increased mortality, delayed development and reduced levels of sex pheromone production^84^.

A GPA2/GPB5 glycoprotein gene was detected in our *C. quadricarinatus* transcriptome. This ancient glycoprotein was proposed to play a role in maintaining ion balance in the midgut of adult mosquitoes *Aedes aegypti*^85^, development and hydromineral balance in *D. meganogaster*, and reproduction in *B. belcheri*^86^. We detected expression of GPA2/GPB5 in both neural tissues tested, and only GPA2 expression in the ovary. It has been suggested previously that GPB5 binds with another element to form a glycoprotein in the ovary of *P. clarkii*^7^. We propose that there may be a stage-specific expression pattern, where GPA2 and GBP5 are expressed differently between maturation stages and later co-express to form a glycoprotein unit. Therefore, a follow-up qPCR study investigating expression levels of GPA2/GBP5 during different maturation stages might be valuable to assess the potential of this glycoprotein relevant to reproduction processes.

### ACP, corazonin, RPCH and the ambiguous scheme for GnRH in crustaceans

ACP is a neuropeptide believed to be the hybrid form of Adipokinetic hormone (AKH) and corazonin^87^. ACP has been demonstrated to act as a neurohormone in the central nervous system of *Rhodnius prolixus*^88^, however we only detected expression of *Cq*-ACP in the eyestalk of *C. quadricarinatus*. Temporal expression profiling shows that both Rhopr-ACP and Rhopr-ACPR are upregulated after ecdysis, and the author suggested that this neuropeptide may be involved in processes associated with post-ecdysis^89^. Still, little is known about the function of this neuropeptide.

Crz is a well conserved neuropeptide that was originally determined to be a cardioactive neuropeptide (and later named after its function)^90^. The role of Crz, however, is not restricted to cardioactivity. We found *Cq*-Crz expression in eyestalk, CNS, as well as heart, thus further supporting the classic role of corazonin as a cardioactive neuropeptide. Crz was recently hypothesized to be involved in regulation of stress responses^91^,^92^, while Crz administration in *M. rosenbergii* has demonstrated inhibition of spermatogenesis^93^. Most recently, Crz was suggested to be the decapod equivalent to GnRH, since the GnRH receptor identified from the ovary of the oriental river prawn *M. nipponense* is a corazonin-like receptor^94^. Still, much work needs to be done to confirm the bioactivity of this neuropeptide in reproduction processes.

In *C. quadricarinatus*, expression of RPCH in the CNS, eyestalk, ovary and gut (relatively low) shows a slightly different pattern from that observed in female mud crab *S. olivacea*, where RPCH was found in the eyestalk, brain and ventral nerve cord, but not ovary^95^, however this might reflect the stage from which the tissues were assessed. Global alignment shows high identity throughout the precursor and strict conservation of the mature peptide. RPCH was shown previously to be an ortholog of AKH in insects and APGWamide in mollusks^96^, where it has recently been shown to promote conditioning and spawning in oysters^97^. Early studies of crustacean RPCH suggested a function in pigmentation^98^, however later studies have proven that RPCH can regulate gonadal maturation in *P. clarkii* as determined by both *in vitro* and *in vivo* experiments^99^,^100^. In these studies, the authors implied that RPCH acts as a neurotransmitter that triggers the secretion of gonad-stimulating hormone, and can therefore induce ovarian maturation^100^. Recently, RPCH was reconfirmed to be involved in ovarian maturation in the mud crab *S. paramamosain*, possibly through a stimulatory effect on the nervous tissues^30^.

Despite numerous reports regarding the absence of GnRH in crustaceans^101^,^102^, there is a trend in discovery of either GnRH-like molecules^14^,^15^,^16^ or GnRH-like receptors^94^. In *C. quadricarinatus* we identified putative transcripts for ACP, corazonin and RPCH, but could not detect any GnRH or GnRH-like transcript in the *de novo* eyestalk transcriptome, perhaps because either (1) the eyestalk does not express these genes, (2) low expression level, or (3) the physiological stage at which the eyestalk RNA was isolated. This result coincides with a recent *in silico* mining investigation that included several decapods, where no trace of a GnRH/GnRH-like transcript could be found^57^. Interestingly, there is an evolutionary connection between GnRH, ACP, RPCH and corazonin at both the receptor and ligand level [(see a canonical review by Roch, *et al*.^103^], supporting the proposition that these factors may share similar functionality.

### Other neuropeptides of interest

In addition to the above neuropeptides that were found in the eyestalk and may have reproduction related functions, we have also identified the following neuropeptides, which can possibly serve other physiological aspects. These include diuretic hormones (DHs), a family of neuropeptides that contributes to the maintenance of water homeostasis in insects. In the current study, two distinct DHs (DH31 and DH44) were detected; DH31 was previously suggested to maintain ionic homeostasis of the hemolymph during ovarian development^5^, while the role of DH44 is still vague. Neuropeptide F (NPF) is an invertebrate NPY-like peptide, sharing high sequence similarity and physiological function with NPY^104^. NPF/NPY maintain a wide range of physiological processes including feeding behaviour, growth, metabolic homeostasis, stress response and reproduction in insects and mammals (reviewed by Nassel and Wegener^104^). For instance, studies have confirmed the role of NPF in regulation of male reproductive processes in the desert locust *Schistocerca gregaria*^105^. It has been shown that ecdysis, an important characteristic of arthropods, is controlled by eclosion hormone (EH)^106^. In insects, the role of EH is somewhat clear, while in crustaceans, despite numerous reports showing EH transcripts in the eyestalk, information on the role of this neuropeptide is still unclear^10^,^107^. Prohormone-4 has previously been characterised in the Eastern spiny lobster (*S. verreauxi*)^9^, *H. americanus* and *C. gigas*^108^. The pre-prohormone was first identified in the honeybee^109^. Still, whether this neuropeptide in crustaceans plays any role in reproduction requires further investigation. In our *C. quadricarinatus* gene expression experiment, prohormone-4 was found in every tissue tested except the ovary. A complete vasopressin-neurophysin precursor is detected from our *de novo* assembly, however the function of this neuropeptide in crustaceans is currently unclear. This precursor was also detected in *M. rosenbergii*^6^. Another neuropeptide detected in the current *de novo* assembly is WXXXRamide, which is a crustacean orthologue of natalisin, a neuropeptide that was proved to be involved in sexual courtship and fecundity in insects^110^, but its role in crustaceans has not yet been resolved.

### Limitations and insights from the current study

The current dataset is still incomplete in characterising the entire *C. quadricarinatus* neuropeptidome, as it is based solely on the eyestalk ganglia. Nevertheless, when comparing the current study with other recent comprehensive neuropeptidome studies in crustaceans^57^, a near-complete representation of the predicted neuropeptides are found, providing a solid foundation for future studies into the functionality of these neuropeptides in decapods.

The spatial expression investigation demonstrated that our predicted peptides can be detected in the CNS as well as the eyestalk ganglia, thus supporting their roles as neuropeptides. Future gene expression analysis can be designed to examine the spatial and temporal expression of these neuropeptides throughout development and in response to processes such as molting and reproductive maturation.

## Conclusion

The current study has provided new information concerning putative neuropeptide genes in *C. quadricarinatus* by mining of eyestalk transcripts, thus building upon accumulating understanding of crustacean neuropeptides involved in key physiological processes. *In silico* data mining combined with RT-PCR experiment resulted in a catalogue of neuropeptides, with an emphasis on those that have been shown to play a role in reproduction in other crustaceans, and likely play a similar role in *C. quadricarinatus*. Results from this study will be useful for both *in vitro* gene-based studies (e.g. design of an effective RNAi experiment to test neuropeptide function) and in applied *in vivo* physiological studies towards control of reproductive processes. This work also lays the foundation necessary for future studies with the aim to manipulating components of the neuropeptidome, which could significantly add to our understanding of reproduction in *C. quadricarinatus* and lead to the development of novel approaches to enhance aquaculture of economically important related species.

## Additional Information

**How to cite this article**: Nguyen, T. V. *et al*. Transcriptomic characterization and curation of candidate neuropeptides regulating reproduction in the eyestalk ganglia of the Australian crayfish, *Cherax quadricarinatus*. *Sci. Rep.* **6**, 38658; doi: 10.1038/srep38658 (2016).

**Publisher's note:** Springer Nature remains neutral with regard to jurisdictional claims in published maps and institutional affiliations.

## Supplementary Material

Supplementary Information

## Figures and Tables

**Table 1:** *De novo* assembly statistics of the reference transcriptome.

	Trinity	CLC
Number of transcripts	195,402	93,455
Total size of transcripts (bp)	94,219,731	63,041,056
Longest transcripts (bp)	9,234	14,169
Number of transcripts > 1 k bp	16,256	13,607
Number of transcripts > 10 k bp	0	6
Mean transcript size	482	675
N50 transcript length	561	746

**Table 2:** Putative neuropeptide precursors predicted in the eyestalk of *C. quadricarinatus.*

Transcript annotation	Transcript size (bp)/ORF size (aa)	Lowest e-value	Closest blast hit	Species
Adipokinetic hormone (AKH)/corazonin-related peptide (ACP)	493/100	3.8e-38	(Veenstra, 2015)	*Procrambarus clarkii*
Allatostatin A (AST-A)	1358/309	1.0e-121	BAE45266.1	*Procambarus clarkii*
Allatostatin B (AST-B)	1525/281	8.0e-63	ALQ28584.1	*Scylla paramamosain*
Allatostatin C1 (AST-C)/Prohoromone 1	1544/104	1.3e-21	XP_014598613.1	*Polistes canadensis*
Allatostatin C2 (AST-C2)	286/36	1.0e-18	AIY69122.1	*Neocaridina denticulata*
Allatostatin CC (AST-CC)	454/136	8.9e-09	BAO00935.1	*Nilaparvata lugens*
BursiconA (partial)	461/53	2.1e-21	ADY80040.1	*Procambarus clarkii*
CCHamide 1	1167/215	6.0e-04	ALM30310.1	*Chilo suppressalis*
CCHamide 2	260/42	1.0e-05	ALM30310.1	*Chilo suppressalis*
Corazonin	1116/111	2.0e-17	ALA65535.1	*Macrobrachium rosenbergii*
DH 31	1020/134	2.1e-57	ACX46386.1	*Homarus americanus*
CRF-DH precursor/DH44	1371/84	7.0e-05	ALG35940.1	*Periplaneta americana*
Crustacean cardioactive peptide (CCAP)	1359/138	1.0e-64	BAF34909.1	*Procambarus clarkii*
Crusteacean female sex hormone-like (CFSH)	854/264	3.2e-79	(Veenstra, 2015)	*Procambarus clarkii*
Crustacean hyperglycemic hormone-1 (CHH-1)	504/131	6.8e-44	BAA89003.1	*Procambarus clarkii*
Crustacean hyperglycemic hormone-2 (CHH-2)	552/135	1.4e-48	AJK31204.1	*Lipopenaeus vannamei*
Crustacean hyperglycemic hormone-3 (CHH-3)	1937/137	2.0e-63	ABA42180.1	*Homarus gammarus*
Crustacean hyperglycemic hormone-like	552/135	6.0e-23	AJK31204.1	*Lipopenaeus vannamei*
Eclosion hormone	1230/82	2.0e-24	ALQ28581.1	*Scylla paramamosain*
Elevenin	600/129	2.0e-03	BAO00952.1	*Nilaparvata lugens*
GSEFLamide	1507/335	2.1e-66	ALQ28590.1	*Scylla paramamosain*
FLRFamide	1248/374	4.4e-74	BAE06262.1	*Procambarus clarkii*
Glycoprotein A2 (GPA2)	861/120	2.1e-50	AKN21239.1	*Locusta migratoria*
Glycoprotein B5 (GPB5)	1321/168	1.6e-45	KDR08334.1	*Zootermopsis nevadensis*
HIGSLYRamide (partial)	724/241	1.3e-07	ALQ28601.1	*Scylla paramamosain*
Insulin-like peptide	743/214	5.0e-157	AIU40992.1	*Cherax quadricarinatus*
Kinin	735/173	5.0e-27	ALQ28594.1	*Scylla paramamosain*
Molt inhibiting hormone (MIH-1)	1331/107	5.0e-72	ACX55057.1	*Cherax quadricarinatus*
Molt inhibiting hormone-like-1 (MIH-like-1)	703/78	5.0e-41	AIZ05253.1	*Procrambarus clarkii*
Molt inhibiting hormone-like-2 (MIH-like-2)	755/107	4.0e-34	P83636.2	*Orconectes limosus*
Ion transport protein (ITP)	533/117	9.0e-40	AIZ05253.1	*Procrambarus clarkii*
Myosuppressin	981/99	1.0e-37	BAG68789.1	*Procambarus clarkii*
Prohormone 4	1101/219	3.0e-97	ALC46992.1	*Drosophila busckii*
Neuroparsin 1	770/105	1.8e-33	AHG98659.1	*Jasus lalandii*
Neuroparsin 2	871/101	2.1e-24	ALQ28570.1	*Scylla paramamosain*
Neuroparsin 3(partial)	368/89	1.03–19	AHX39208.1	*Metapenaeus ensis*
Neuropeptide F1	468/87	1.4e-17	AEC12204.1	*Litopenaeus vannamei*
Neuropeptide F2	470/104	6.0e-24	ALQ28586.1	*Scylla paramamosain*
Orcokinin	900/187	4.9e-70	ACD13197.1	*Homarus americanus*
Pyrokinin	1418/201	5.7e-32	ALQ28575.1	*Scylla paramamosain*
Pigment dispersing hormone 1 (PDH-1)	509/79	1.0e-27	JC4756	*Penaeus sp.*
Pigment dispersing hormone 2 (PDH-2)	393/79	3.0e-14	JC4756	*Penaeus sp.*
Pigment dispersing hormone 3 (PDH-3)	393/79	6.0e-24	JC4756	*Penaeus sp.*
Proctolin	697/96	3e-04	AEX08669.1	*Rhodnius prolixus*
Red pigment concentrating hormone (RPCH)	420/90	3.8e-43	AAV80404.1	*Cherax quadricarinatus*
Ryamide	462/131	1.0e-10	NP_001280530.1	*Tribolium castaneum*
Short Neuropeptide F	1617/127	5.3e-39	ALQ28574.1	*Scylla paramamosain*
SIFamide	619/75	3.8e-24	Q867W1.1	*Procambarus clarkii*
Sulfakinin	476/116	2.4e-49	ABQ95346.1	*Homarus americanus*
Tachykinin	1139/235	8.4e-100	BAC82426.1	*Procambarus clarkii*
Trissin	1052/200	2.0e-09	NP_650471.2	*Drosophila melanogaster*
Neurophysin -Vasopressin	577/154	1.8e-28	ALQ28600.1	*Scylla paramamosain*
WXXXRamide (partial)	238/79	2.0e-11	ALQ28592.1	*Scylla paramamosain*
