# Supplementary material for: Transcriptomic characterization and curation of candidate neuropeptides regulating reproduction in the eyestalk ganglia of the Australian crayfish, Cherax quadricarinatus
Source: Sci Rep. 2016 Dec 7;6:38658. doi: 10.1038/srep38658 (PMC5141488; doi:10.1038/srep38658)

Transcriptomic characterization and curation of candidate neuropeptides regulating reproduction in the eyestalk ganglia of the Australian crayfish, *Cherax quadricarinatus*

Tuan Viet Nguyen[a], Scott F. Cummins[a], Abigail Elizur[a], Tomer Ventura[a,*]
[a] *GeneCology Research Centre, Faculty of Science, Health, Education and Engineering, University of the Sunshine Coast, Sunshine Coast, Queensland, Australia*.

Supplementary file 1 - Neuropeptide aa sequence

>AKH/corazonin-related peptide (ACP)

MVAWQVMLAVVCLAIAPTMAQITFSRSWVPQGKRSGSGGSLVNAPGAPDLTIDPCRDVRLTTLTQVASHLVELM
DDASEGTQDDALRLKHALVARRQRML

>Allatostatin A

MDLDKRPRNYAFGLGKRDPDLDLDKRPRNYAFGLGKREPDMDLYKRPRNYAFGLGKRTSDEEEDDDDEQYYPYG
LRKRPRIYSFGLGKRSLGVADDYDELNDEEEDEELGDFEQYGEDLKRTASYGFGLGKRGNVLSNGFGRRSYDFGLGK
RAAGQYAFGLGKKANPYSFGLGKRAGPYAFGLGKRSGPYAFGLGKRAGPYAFGLGKKVDPYAFGLGKRSGQYAFG
LGKKSGQYSFGLGKRSGPYSFDLGKNVDYDSSDAYTLGRRSGTYSFGLGKRAGPYNFGLGKREASDDAHQEENREP
VAEQASS

>Allatostatin B

MQHVRGAWTLLVLVALVQLVVSQEEVTPDHSDDKRVGWSSMHGTWGKRPDLEDTQLEVAEDKRTNWNKFQG
SWGKRGDDLADAELQAAEDKRTNWNKFQGSWGKRGDDFTDADLQDAALDKRTNWNKFQGSWGKRGDXXTG
AVFRELGARGRGRTCKGHGASALRTTPQMMTFTTTPPPRRATXXLVSDEAQDISSMALARMMASAVPQKRGWT
LWGKRPVNTRVSPRSTNWSSLRGTWGKRSADWNKLRGAWGKRADWDQFRGSWGKRVPGALSEATPQA

>Allatostatin C1/Prohormone 1 (Trinity)

MLTRCVSLVTVALLALVAVSQVSGKALPDQSSQAYPEPQHMLDPYGNHLVDDDGSLDAALINYLFAKQMVERLR
NNADIKDLQRKRSYWKQCAFNAVSCFGKRK

>Allatostatin C2

KRMFVPLSGLPGELPTIKRQIRYHQCYFNPISCFRR

>Allatostatin CC

MLGRSSISITSLLLSLLLVVSTEARVPQVASRPQYLEVVRPVMPNVPLEPLGLTQGVNQQIAETVSTPRKRAAIVLDKL
MYALQKALDDNPATSPASTRSPPPFYRSRTYTGQMDLQRRGNGDGRLYWRCYFKIVS

>BursiconA

KLWQTERSCMCCQESGEREASVILNCPNARQGEPKQKKVSVQVNIIMQDKSLTLKIA

>CCHamide 1

MVFPRSSTLLLLVFPVILLLCFPPSSAHRVLKGGCLNYGHSCLGAHGKRASAPPHRPLVPRPLLDILDALTTPTRTNNL
YAHTSDNSMREPRTRNPEGRFLAPSAGNQLADLGLDLRADGLMDDINEEVDIMGGISGRGGRMGGSDERGERL
GGSSEVDPDAVLYYGTLDDYNDVRYKRQASPGSAAVTKRLNTWAYSPLQQEEEQEEEEKEEEE

>CCHamide 2

MAGAALTFVVLVVGVVTLSSQAWGSCSQFGHSCFGAHGKRDG

>Corazonin

MEKRNSQVVLMVVLVVALTVSLTAAQTFQYSRGWTNGRKRSDPSVGVVRDVTDLLADSTHRLPSHRPLPPTHAL
PKNLEERLRALELGLNAALKASATFPPAADDQYYSDN

>Diuretic hormone 31/prepro-calcitonin-like diuretic hormone

MNSTGAVFVSLLVTLVLVSTVSSATLNREARAVVEIDDPDYVLELLTRLGHSIIRANELEKFVRSSGSAKRGLDLGLGR
GFSGSQAAKHLMGLAAANFAGGPGRRRRSPDNALDLSHDDNLYAQDQADLTESSR

>CRFlike diuretic hormone (DH44)

MAKRTWPNAFPRRRTSGLSLSIDASMKVLRQALYLEMARKKQRQHLQRAQHNQKLLNDIGKRDVTRQLQQERPS
AEQQRQQQRN

>Crustacean cardioactive peptide (CCAP)

MSSVCWYGRAGVLMTAALLFFVLVAHTNAVPLAKRDIGDLLEGKDKRPFCNAFTGCGKKRSDPGVEGVASSSELD
ALAKHVLAEAKLWEQLQTKMEVMRSLAARMENNPLYRRKRSLPHQPRHNLTSTPKQKVNSKKQ

>Crustacean female sex hormone like

MAFLTVCDHQSRLFYRCFQAQGRGRSAMLSLAAVHLLVTALSLSVSVASGSEVALANTFKNDGEDGQVGVSSWA
LPQWWWLTAVLPLPQSQPRYHTSHSTTKTASDLATLSEEDSAMSIVLPGEQLGQSEGGNSEVLQATTPAEGRLNK
RSHLCRSPRCHRGMTNIIPASEVKQSWRKEYLSVPEALVQFSQVQAEEEVCKDLSVQLYSVDLTEHYLEPLWVRETV
HLGMCPSKLQTRHLGENVWPPNVVEIKCLCQRETCSNL

>Crustacean hyperglycemic hormone 1

MTSCRTMWSLVVVAMVVVVVTLGPTGVRGRSVEGSVRLERLLSAGSSSAPLGFLSQDHSLNKRQVFDQACKGVY
DRALFKKLDRVCDDCYNLYRKPYVAASCRENCYSNLVFRQCLDDLLLVDVVGTPCYC

>Crustacean hyperglycemic hormone 2

MTSCRTMWSLVVVAMVVVVVTLGPTGVRGRSVEGSVRLERLLSAGSSSAPLGFLSQDHSLNKRQVFDQACKGVY
DRALFKKLDRVCDDCYNLYRKPYVAASCRENCYSNLVFRQCLDDLLLVDVVDEYVSGVQIVGK

>Crustacean hyperglycemic hormone 3

MVAC

RMMWSLVVVAMVVVVVTLGPTGVRGRSVEGSVRLERLLSAGSSSAPLGFLSQDHSLNKRQVFDQACKGVYDRAL
FKKLDRVCDDCYNLYRKPYVAASCRENCYSNLVFRQCLDDLLLVDVVDEYVSGVQIVGK

>Crustacean hyperglycemic hormone-like

MSHCAVTLVVVLVVLTTLCSGRSLSSGEVQWLLQTSPYKSLPQEYNVRRRDSGLEYHSVTKRAVFDSQCKGFYDRG
VWARLNRVCVDCQNLYRSPEIENECRMGCFATKYFTSCVSNLLLPVDEYQDMAALVRGS

>Eclosion hormone

MSFKPDMRVVVLSVMFLMALVTLSQAASITSMCIRNCGQCKEMYGDYFHGQACAESCIMTQGVSIPDCNNPATF
NRFLKRFI

>Elevenin

MAATARMCLGLPMVVLLVSLACLVAQSHAVDCRKFVFAPVCRGIIAKRMITEKRSSFRPAAAADTQWNAQYGVP
TQTDTNDLLLAPSYEEVMEPRTQDDIVVVRAGEDVVHVPAYVYEIIERSLQGERK

>GSEFLamide

MVRYWPCLVVPCVLVCCWCALSTALPTSFLRPDDPAEVVVKRMAGAPNESMLRYILMAISNPAARYQSPQLLNR
GVRRIGSEFLGKRSAGRLTAADNPGNFVSENTSNEDGTEKDLKKEQFSFTGQYDYDDRAAENPSEDLFTAKPKKSV
RGFHADNSYEGLKNFFGMLTSKKMGSEFLGKRMGSEFLGKRVVDSEYLGKRAMGSEFLGKRAMGSEFLGKRAM
GSEFLGKRAMGSEFLGKRAMGSEFLGKRAMGSEFLGKRTMGSEFLGKRAMGSEFLGKRAMGSEFLGKRAMGSE
FLGKRVMGSEFLGKRLYGPEFVRALEYDQKRAVGSEFLG

>FLRFamide

MTRSYVMLLEATMVVATLCWSAHAQAAYVPPSGAAVSVASQDLDGPSSGEDMVGKPEKRLLKYFLPGSQAWLS
GVEGLYPTRQEGTKRGYSDRNYLRFGRSDEDKRGPSRNFLRFGRSGLGDYSSSSGDEELIDSVEKRGRNFLRFGRDP
SRNFLRFGRSDMEEFGLAGGPVEFPSGVQDELNDLEFPIDEKRDGHSTYIRYLKGNRDFPRFGRGDRNFLRFGRSV
DRQKSSMSFENCDEEPKTHDVTSTPSPTPVQPMTRTKQQAATHSIASSDSDKTTSHRMKRNVSRVYGYITLPTHTR
DINPEEDAINVAYSDEPQVVDKRGYNKGFLRFGRDRNFLRFGKRDVSGEYPTYSSISSSESSGSLLGERPAR

>GlycoproteinA2 (GPA2)

MVKVWVVLVTCLVVSTTSYKHAWQTPGCHKVGHTRRISIPECVEFDITTNACRGYCESWSVPSAWQTLVYNPHQ
VVTSIGQCCNIMETEDVKVRVMCIEGPRELVFKSASTCDCFHCKKY

>GlycoproteinB (GPB5)

MVTGGGRSGSVSGGARGSVGGRVVAMVAVGAALLALLLPAAAINPQSTLECHRRQYTYKVHKSDDDGRVCWDF
VNVMSCWGRCDSNEIADWKFPYKRSHHPVCMHEQTQLTEVTLRHCDDGAAPGTEMYSYHEAARCACSVCKSSE
ASCEGLRYRGARRAPRADVPRG

>HIGSLYRamide (partial)

EFEEEKRHLASLYKPRRSDNFLDDFDEEKRHLASLYKPRRNDEFLNNFIEEKRHLASLYKPRRNDESLEEFEEEKRHLA
SLYKPRRNDNSLDEFEEEKRHLGSLYKQRRNDESLDEFEEEKRHLGSLYKPRRNDNSVNEFEEEKRHLASLYKPRRSD
SSVNTLDEEKRHIGSLHKPRRSDDSLDTFDKEKRHLSSLYKPRRNDNSLDTFEEEKRHIGSLYKPRRNDGSQDTFDEE
KRHIGS

>Insulin-like peptide

MLALTAMFVLGSTSWALESDLIRQIESRTETEWQTLWSKERLSLCRARLRHNLDTICGKDVYRRSLAPPRPAPYHHI
FKRRTDICLQVHDTGGARRVEGEKHLSKSSNRVKRVREVLVNLSPDIIQTSPATDTGQPSVQDRHVHSRYRSPFLSV
HQANLFVTTWVRDHQGRHYRRRRQSSSITAECCTTTGCTWEEYAEYCPTSSRLRAGVALI

>Kinin

MFXNKAIMAWIGKNLPTTQLTMADKRAFSAWAGKRSSDHLDGQITNEVTKFSPWAGKQLEETIPGDNDLAVRTI
NDEDSLSDDQKRSSFSAWAGKRSDDEKRQSFSAWAGKRSHNDEKRDNFSEWVGKQNDNDKRQQAFSAWAGK
RNDNNEKRQ

>Molt-inhibiting hormone-1

MVKHATQSCSALRPWLIVMVVGLLVHQTTPTLTDDCPGAMGNRHIHTMLLRVCGDCYNVLRDPEIEVDCRSGCF
TSDTFKSCLELIERGDEFFDFMRRVGILNAGGK

>Molt-inhibiting hormone-like-1

MGNQKSECFYARRVWLLVLIALVVQQSTARFIEDDCPGVVGNRNVHSMVMRVCEDCYNVFRHPEVAVGCSVRK
STQTS

>Molt-inhibiting hormone-like-2

MGNQKSECFYARRVWLLVLIALVVQQSTARFIEDDCPGVVGNRNVHSMVMRVCEDCYNVFRHPEVAVGCRRAC
FSSKMFKSCLSALQREEEYPDFLRLIGILNAGRK

>Iron transport protein

MSIFQVCTLGRMCLWFLLIIGLLSQSQQGAAHFYKIQPGTFKEFQYINCQGTYNRSTYTKLIRICEECQNLYRNDYTV
SLECKENCFQNEMFDKCVLSLLLAHKEEEYKNMITYASG

>Myosuppressin

MMFRGSSWCSVVLVGVVVALVVCVGVGEAIPPPICLNQQLPLSAYAKKLCVALANISEFSRAMEEYLDAQAIKNS
MPVNEPEVKRQDLDHVFLRFGRSQ

>Prohormone-4

MGRHSSSSVSQGSALVLVLLAATASAIDISRLPGHRHYTKRSAYGGYGYKHHDYSHQAPAERECMAYEPFRCPGG
QVCISIQYLCDGAPDCPDGYDENPKLCTAAKRPPVEETASFLQSLLASHGPNYLEKLFGSKARNALQPLGGVEAVAV
ALSESQTIDSFGDTLHLMRSDVEHLRSVFMAVENGDIGMLKSLGIKDSELGDVKFFLEKLVNTGFLD

>Neuroparsin 1

MKISCSSNVYIFLAYCSLLLLLLLQNTVASPICPERNEITEEDLNKCKYGVVLGWCGNAVCGKGPAETCGGWWDEN
GICGEGMYCVCGYCAGCTTTLECALGRFC

>Neuroparsin 2

MRTLTFFISFVVTFYCLVLLFQEAAAAPRCNTHDRPPPSNCKYGTVRNWCRNGVCAKAPGESCGGHWYEHGKCGI
GTFCLCGVCIGCSTIDGRCADGPLMC

>Neuroparsin 3

CVLLCREAAAAPRCNRQGTRTPSLNCKYGTVADWCGNRVCAKGPGETCGGDWGEKGACVAGTYCSCGFCSCGY
CTGCAANLDCWFGHFC

>Neuropeptide F1

MYRQMLSALLVGVVVVGVLEMGVADAKPAPPQVAAMADALKYLQELDKYYSQVSRPRFGKRSEYVAPADDTM
MEASERILESLSRRR

>Neuropeptide F2

MRGTMMIGAVVAVMVAAVVAGRMSAARADTSAVEALQAMHEAAMAGILGSAEIQYPNRPSMFKSPVELRQYL
DALNAYYAIAGRPRFGKRGNHGPQRTEELDDY

>Orcokinin

MTRESHSQSHAAMSSQVLSLLLLLSFTALAAAGTIKAAPARPSTQQHDATFTDGAPVKRFDAFTTGFGHSKRNFDE
IDRAGFGFAKKNFDEIDRSGFGFNKRNFDEIDRSGFGFNKRNFDEIDRTGFGFHKRDYDAFLDKRNFDEIDRSGFGF
VKRVYVPRDMANLYKRNFDEIDRSGFGFVRRNAE

>Pyrokinin

MDEEMWPSPVLSEEEVATAAGSPDKRADFAFSPRLGKKADFAFSPRLGKKADFAFSPRLGKKADFAFSPRLGKKA
DFAFSPRLGKKADFAFNPRLGKRADFAFRPRLGKKADFAFSPRLGKKADFAFSPRLGKKADFAFSPRLGKKADFAFN
PRLGKKADFAFSPRLGKREREEDSGEREPSRTQAYISRPSRPYFSPRLG

>Pigment dispersing hormone 1

MRSATMLALLVAVIMSAAISEAQELKYPEREVVAELAAQILRVVHGPWSTVVPGPHKRNSELINSILGLPKVMNDA
GRR

>Pigment dispersing hormone 2

MRGVVVVVVLVMVGMLSILTQAEGLKSQEREVVAELAAYILRVVHGPLNLYADLPSKRNSEILNTLLGSPTLMSEV
GKR

>Pigment dispersing hormone 3

MHSSVMVVVLMMLVMATVFTQAQDLKYPERQVVTELAAQIMRVAQGPWSTSLDLPTKRNSGLINSLLGIPKVM
NDAGRR

>Proctolin

MARSGMLVVMALVVLVAALTNARYLPTRADDSRLEEIRELLREVLERTADGGSSISSSSSRVSGSGYDKRFLFKRAA
AAEGGVAGEVVEPLLNLPQ

>Red pigement concentrating hormone (RPCH)

MVRGSVALLLVVLVASSCVSAQLNFSPGWGKRTGTAAGGPDQTILHSSSPAAVASDNCGTIPVSAVMHIYRLIRTE
AARLVQCQEEEYMG

>Ryamide

MSRALCPALVILAALLALVASQGFYSQRYGKRGETREMAVRSVRYANQNARSNLPQGLPEIKIRSSRFIGGSRYGKR
SGSPSEPELPSVMTPEGEDTEVAATLLLGDSILCFLVDVPDIYRCLRKPTSEEA

>Short Neuropeptide F

MAVSVVKCWVSLVCCCLLLAHLNTAVPTPPDYDALSEVYNWLSDHGLERRAPPSMRLRFGKRDMGWQVAQRS
MPSLRLRFGKRTLDQGDALFDHDLVRKDSRIPALRLRFGKRDSYGQEEDMASQEQ

>SIFamide

MSVQTRMVVVVAVLMVVLAVLSHPVSAGYRKPPFNGSIFGKRSGDVVYEPGKALASACQVAVEACAAWFPQTE
KK

>Sulfakinin

MKWTSWCAAILAVMAAVLLCGGVTAPARPSSLARVLAPVVRHRLEEGGLAPALVEELVADFEDPELLDFRDAAGK
REFDEYGHMRFGKRGGDYDDYGHLRFGR

SLHSHKNQHSIFH

>Tachykinin

MVRAWSWAAAVGVIVAVSVVGAVGGDGQESNERERRAPSGFLGMRGKKDLSSHLVDTTASNQYSLHDSYPAA
AAAAAALYGLRDDSGPMVLAVPWRAKKAPSGFLGMRGKKSDEEVFGEAGYHSDLETLLKRAPSGFLGMRGKKAP
SGFLGMRGKKAPSGFLGMRGKKLYDDDSEMDAYIQALTAMVEGEQKRAPSGFLGMRGKKSPYGVTTDEEMED
MASLDKRAPSGFLGMRG

>Trissin

MHYLSIFLAWAVVVGGTRAWSSSEVSCASCGSECQSACGTRNFRACCFNFQRRRRSDSLAHSSTSMTEADYLGLQ
GLLKPGVSSTRDSHLSQVLRSLSRTLDGSAPDPLFYKDPSESLSSVLASLMQESNEEEEEESDLATSRGDTDGHVNSG
GYSGTFGDDSALSRLVALTLHRPPPALHQRQQTHHQRYSSSPTNIRK

>Vasotocin-Neurophysin

MQGGVGVVMVVGVLVGAATACFITNCPPGGKRSGHTTQLAHFRTCTSCGPGLRGRCMGPEICCGPGLGCFLGT
REARMCRSENLVPLTCTNTDLNTCGRMREGRCAASGICCTEMRCEFDSSCLMEGREGSVEEQPIERQRLAILPSLS
DIQWS

>Natalisin/WXXXRamide

WIARGKKVVVHPFWVARGKKEVNPFWVTRGKEEAAARPFWIARGKKEVHPFWVARGKKEEESHPFWVARGKK
GETNPFW

Supplementary file 2 – Genbank accession number

Neuropeptide	*Species*	Accession number	Note/comments
ACP	*P. clarkii*	Veenstra, 2015	
	T. castaneum	NP_001159497.2	
	N. lugens	BAO00933.1	
	T. castaneum	EFA12888.1	
Corazonin	*P. clarkii*	Veenstra, 2015	
	S. verraeuxi	Ventura et al, 2014	
	M. rosenbergii	ALA65535.1	
	N. lugens	BAO00944.1	
	B. mori	NP_001036899.1	
GnRH	*C. gigas*	ADZ17180.1	
	R. norvegicus	XP_006252158.1	
	P. marinus	AAF78456.1	
	O. vulgaris	BAB86782.1	
	H. sapiens	AAI26438.1	
	G. gallus	AET50997.1	
CHH	*H. americanus*	P19806.3	
	O limosus	Q25588.1	
	M. japonicus	BAA13481.1	PENJP_CHH-1
	M. japonicus	Q9U5D2.1	PENJP_CHH-2
	L. vannamei	AAN86057.1	
	M. rosenbergii	Sawaros et al, 2015	

	M. rosenbergii	Sawaros et al, 2015	
	M. rosenbergii	Sawaros et al, 2015	
	P. clarkii	AAB25534.1	CHH-1
	P. clarkii	AIZ05252.1	CHH-2
	P. clarkii	BAA89003.1	CHH-3
	P. clarkii	Veenstra, 2015	CHH-4
	M. japonicus	ABN11282.1	PENJP_CHH-like
	M. japonicus	O15980.1	PENJP_CHH-1
	M. japonicus	Q9U5D2.1	PENJP_CHH-2
	M. japonicus	Q94676.1	PENJP_CHH-3
	M. japonicus	P55847.2	PENJP_CHH-4
	M. japonicus	O15981.1	PENJP_CHH-5
	M. japonicus	P81700.1	PENJP_CHH-6
	M. japonicus	O15982.1	PENJP_CHH-7
	P. monodon	AAQ24525.1	PENMO_CHH-1
	P. monodon	AAQ24526.1	PENMO_CHH-2
	P. monodon	AAQ24527.1	PENMO_CHH-3
	P. monodon	AAC84145.1	PENMO_CHH-4
	P. monodon	AAC84146.1	PENMO_CHH-5
	J. lallandi	P56687.1	
	M. lanchesteri	O77220.1	
	N. norgevicus	AAQ22391.1	
	L. schmitti	P59685.1	
	O. limosus	Q25588.1	
	H. gammarus	ABA42180.1	
MIH	*M. rosenbergii*	AAL37948.1	
	M. rosenbergii	AAL37949.1	
	P. clarkii	Veenstra, 2015	
	L. vannamei	AAC60516.1	LITVA_MIH-like
	L. vannamei	AAR04348.1	LITVA_MIH-1
	P. monodon	AAR89517.1	PENMO_MIH-1
	P. monodon	ACS88073.1	PENMO_MIH-2
	J. lallandi	P83220.1	JASLA_MIH
	L. stylirostris	AAL55257.1	
	M. japonicus	BAE78494.1	
VIH	*H. gammarus*	ABA42181.1	
GIH	*H. americanus*	P55320.1	
	N. norvegicus	AAK58133.2	
ITP	*S. gregaria*	AAB16822.1	
	L. vannamei	ABN11282.1	
	P. monodon	BAB69829.2	
Neuroparsin	*B. mori*	XP_012549557.1	
	S. paramamosain	ALQ28589.1	NP-3
		ALQ28571.1	NP-4
		ALQ28588.1	NP-2

		ALQ28570.1	NP-1
	M. enis	AHX39208.1	
	J. lallandi	AHG98659.1	
	P. clarkii	Veenstra, 2015	NP-1
	P. clarkii	Veenstra, 2015	NP-2
	P. clarkii	Veenstra, 2015	NP-3
RPCH	*P. clarkii*	Veenstra, 2015	
	S. verraeuxi	Ventura et al, 2014	
	M. rosenbergii	ABV46765.1	
	S. paramamosain	AGW45011.1	
	C. sapidus	AAF21244.1	
	C. maenas	AAB28133.1	
Vasopressin-neurophysin	*P. clarkii*	Veenstra, 2015	
	M. rosenbergii	Sawaros et al, 2015	
	D. pulex	EFX71881.1	
	D. rerio	AAO11656.1	
	S. paramamosain	ALQ28600.1	
	H. saltator	EFN79183.1	

Supplementary file 3 – Primers used

Neuropeptide gene	Left primer	Right primer
CCAP	GCGTGCTGATGACTGCTG	TGGGTGTTGATGTGAGGTTG
CFSH	GCTACCACACCTCGCACTC	CCTTACACACCTCCTCCTCTG
GPA2	TCCTGTTAGTGGTGTTCTCGTC	GGTGTGGGTAGTGTTGGTGA
GBP5	CCCTCACACACAACAAGCAG	GACGAAATCCCAGCACACC
NP-1	GGCTCACCTACCACCCACT	CCGTTCTCATCCCACCAG
Np-2	GAGGAGGTTGTGAAGGGTGA	CTTGGTGTCGTCAGCAGGTA
NP-3	GACCCCATCTCTCAACTGC	GACCGAACCAACAATCCA
SIF	GAATGGTCGTGGTTGTGG	GTGTTGCTGGCTGTGAAGTC
RPCH	AAGTCTCGCTGCTTCAGTCC	CTCCTGCTGGTGGTACTGGT
ACP	ACATCTCTCCCCCTCGATCT	AGACTGCCATAGTGCCTGCT
CRZ	TGGACGAATGGACGGAAG	CAAAGTGAAACGGAAGATGC
Pyrokin	AGGAGGAGGTAGCAACAGCA	GGGGGAGAAATAAGGTCGAG
Myosuppressin	AGCGCTCCTCTCGTACACAC	GAGCCCCTTCGTCTTCACTT
Prohorme-4	TGATGAGAACCCCAAGCTGT	CAAGGAAGCCAGTGTTGACC

Supplementary file 4 – Sequence reads statistics

Raw reads statistics

Total number of sequences: 156268765

Sequences flagged as poor quality: 0

Sequence length: 90

%GC: 40

Per base sequence quality Cq_eye_1.fastq (Forward reads)

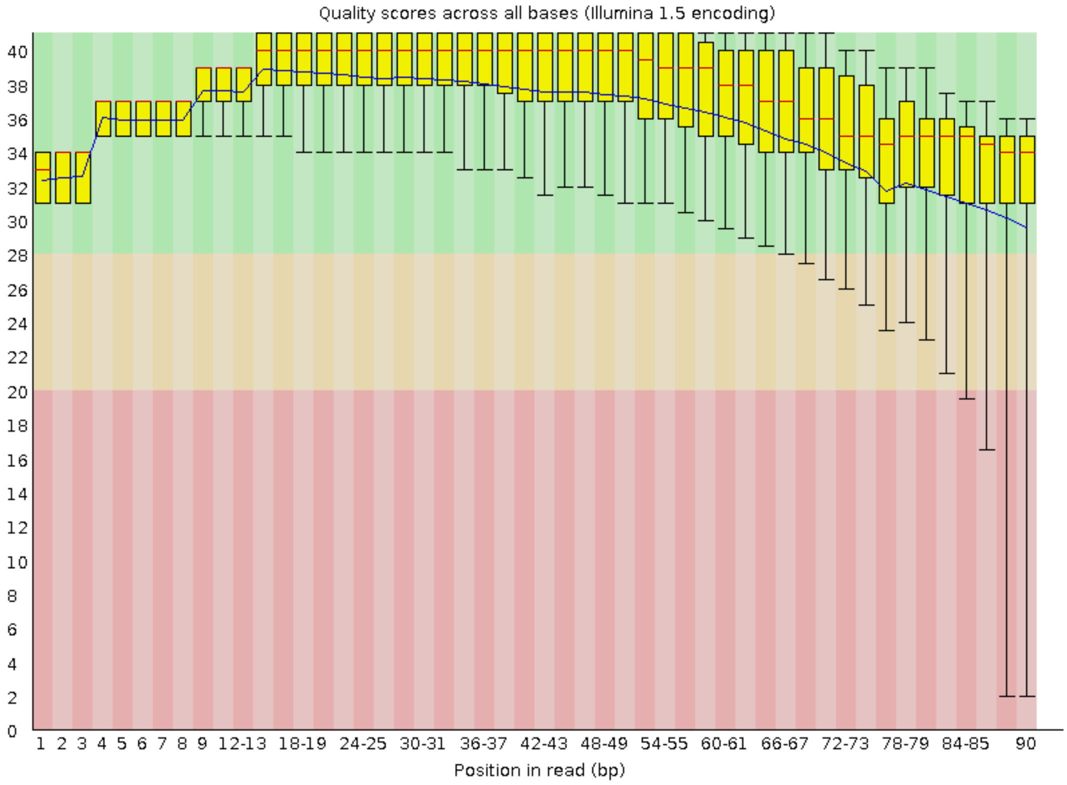

Per base sequence quality Cq_eye_1.fastq (Reverse reads)

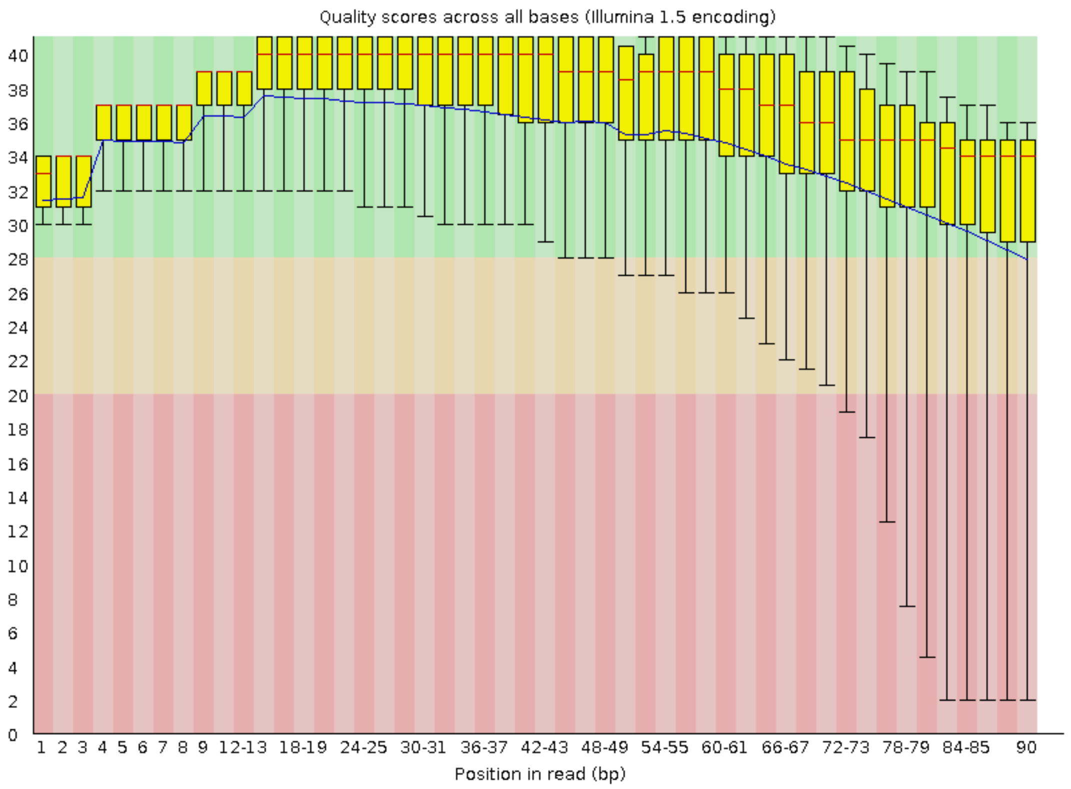

Quality scores across all bases (Illumina 1.5 encoding)
40
38
36
34
32
30
28
26
24
22
20
18
16
14
12
10
8
6
4
2
0
1 2 3 4 5 6 7 8 9 12-13 18-19 24-25 30-31 36-37 42-43 48-49 54-55 60-61 66-67 72-73 78-79 84-85 90
Position in read (bp)

Supplementary file 5 – Overview of neuropeptide detected in related species

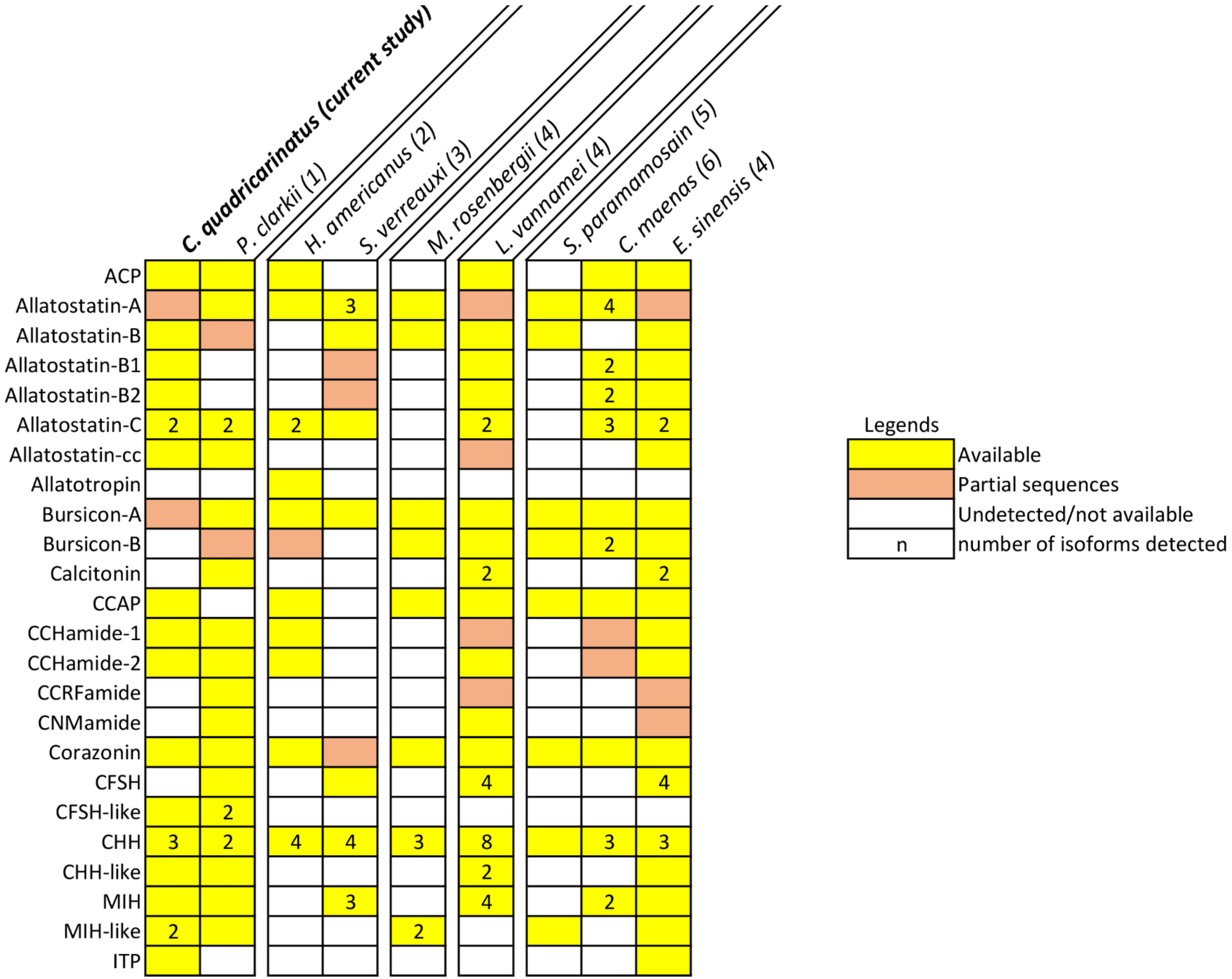
C. quadricarinatus (current study)
P. clarkii (1)
H. americanus (2)
S. verreauxi (3)
M. rosenbergii (4)
L. vannamei (4)
S. paramamosain (5)
C. maenas (6)
E. sinensis (4)
ACP
Allatostatin-A
Allatostatin-B
Allatostatin-B1
Allatostatin-B2
Allatostatin-C
Allatostatin-cc
Allatotropin
Bursicon-A
Bursicon-B
Calcitonin
CCAP
CCHamide-1
CCHamide-2
CCRFamide
CNMamide
Corazonin
CFSH
CFSH-like
CHH
CHH-like
MIH
MIH-like
ITP
Legends
Available
Partial sequences
Undetected/not available
n
number of isoforms detected

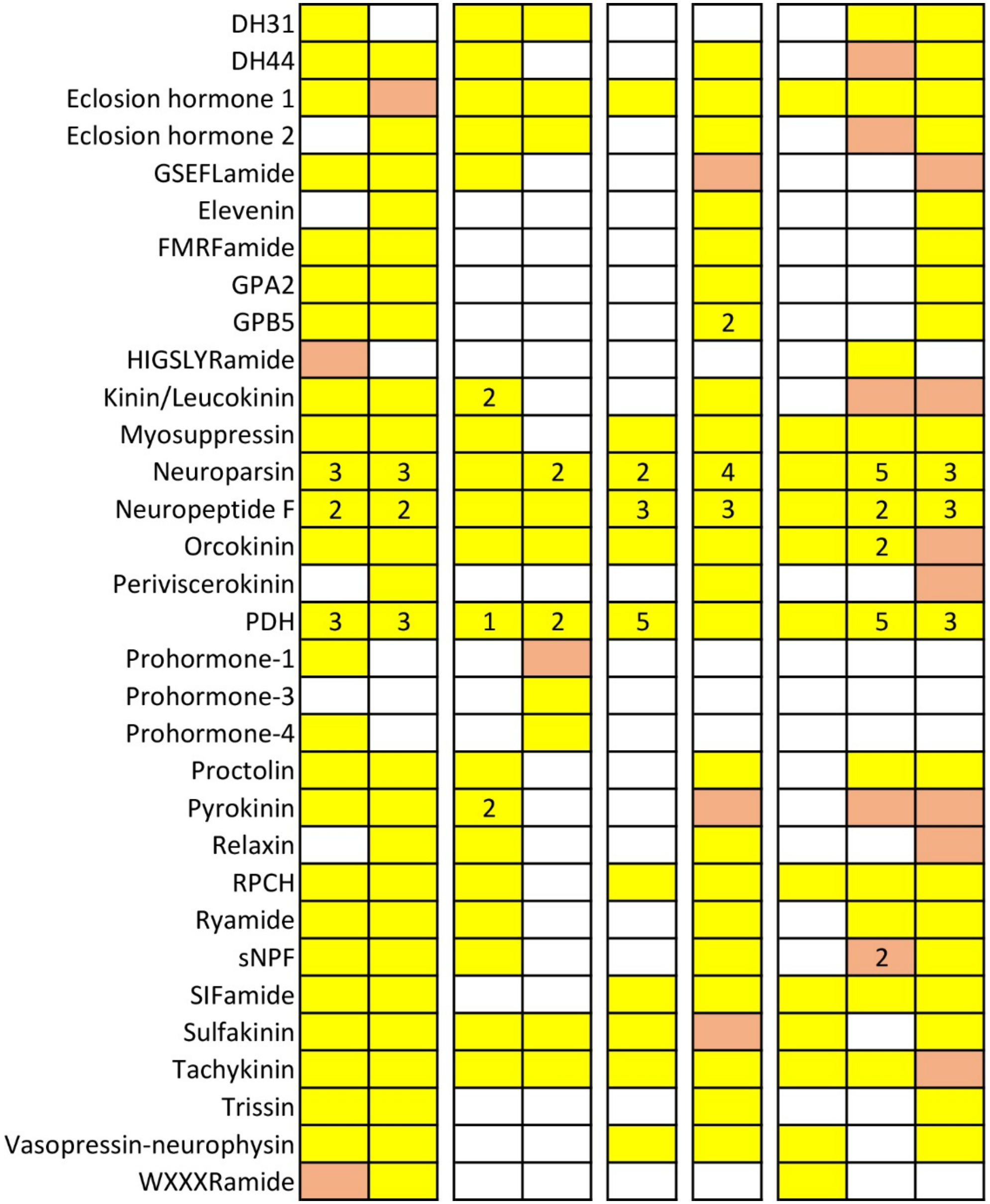
DH31
DH44
Eclosion hormone 1
Eclosion hormone 2
GSEFLamide
Elevenin
FMRFamide
GPA2
GPB5
HIGSLYRamide
Kinin/Leucokinin
Myosuppressin
Neuroparsin
Neuropeptide F
Orcokinin
Periviscerokinin
PDH
Prohormone-1
Prohormone-3
Prohormone-4
Proctolin
Pyrokinin
Relaxin
RPCH
Ryamide
sNPF
SIFamide
Sulfakinin
Tachykinin
Trissin
Vasopressin-neurophysin
WXXXRamide

1 Veenstra, J. A. The power of next-generation sequencing as illustrated by the neuropeptidome of the crayfish *Procambarus clarkii*. *General and comparative endocrinology* **224**, 84-95, doi:10.1016/j.ygcen.2015.06.013 (2015).
2 Christie, A. E. *et al.* Neuropeptidergic signaling in the American lobster *Homarus americanus*: New insights from High-Throughput nucleotide sequencing. *PloS one* **10**, doi:10.1371/journal.pone.0145964 (2015).
3 Ventura, T., Cummins, S. F., Fitzgibbon, Q., Battaglene, S. & Elizur, A. Analysis of the central nervous system transcriptome of the eastern rock lobster *Sagmariasus verreauxi* reveals its putative neuropeptidome. *PloS one* **9**, doi:10.1371/journal.pone.0097323 (2014).
4 Suwansa-ard, S. *et al. In silico* neuropeptidome of female *Macrobrachium rosenbergii* based on transcriptome and peptide mining of eyestalk, central nervous system and ovary. *PLoS ONE* **10**, e0123848, doi:10.1371/journal.pone.0123848 (2015).
5 Veenstra, J. A. Similarities between decapod and insect neuropeptidomes. *PeerJ* **4**, e2043, doi:10.7717/peerj.2043 (2016).

Supplementary file 6 – Full-length gels which have been cropped in the main text

PCR results in Figure 12 of the main text, red lines represent cropping lines. All gels have been run under the same experimental conditions. 1. *ACP*, 2. *Corazonin*, 3. *CCAP*, 4. *CFSH-like*, 5. *GPA2*, 6. *GBP5*, 7. *Myosuppressin*, 8. *NP-1*, 9. *NP-2*, 10. *NP-3*, 11. *Prohormone-4*, 12. *Pyrokinin*, 13. *RPCH*, 14. *SIFamide*, 15. *Beta-actin*

1. 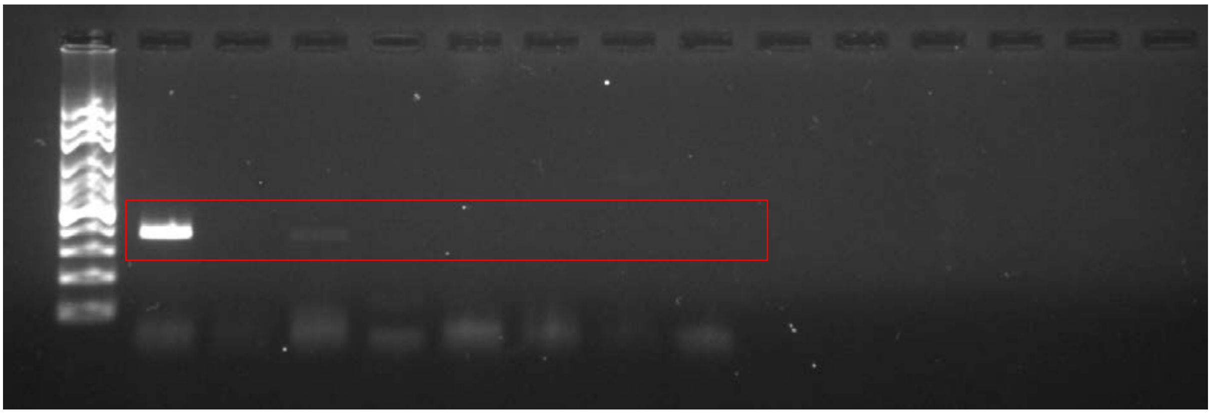

2. 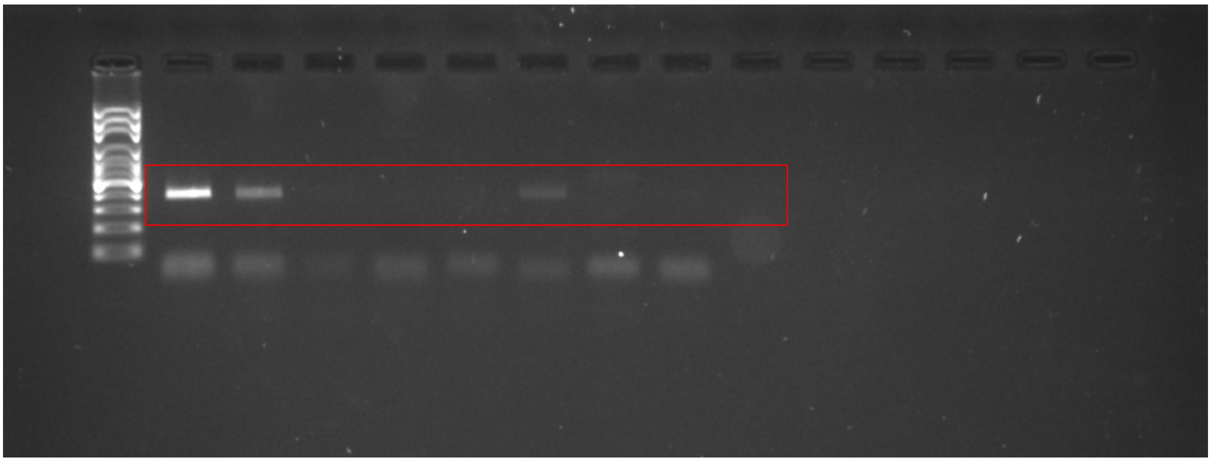

3. 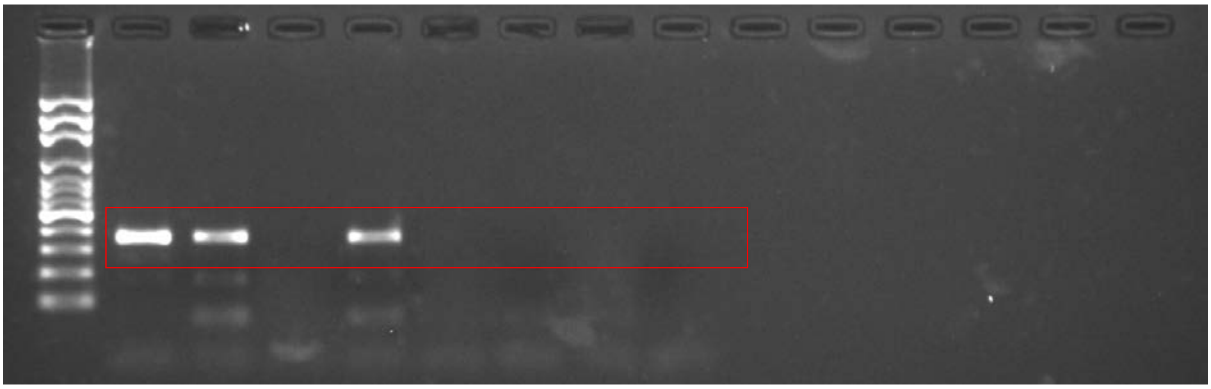

4. 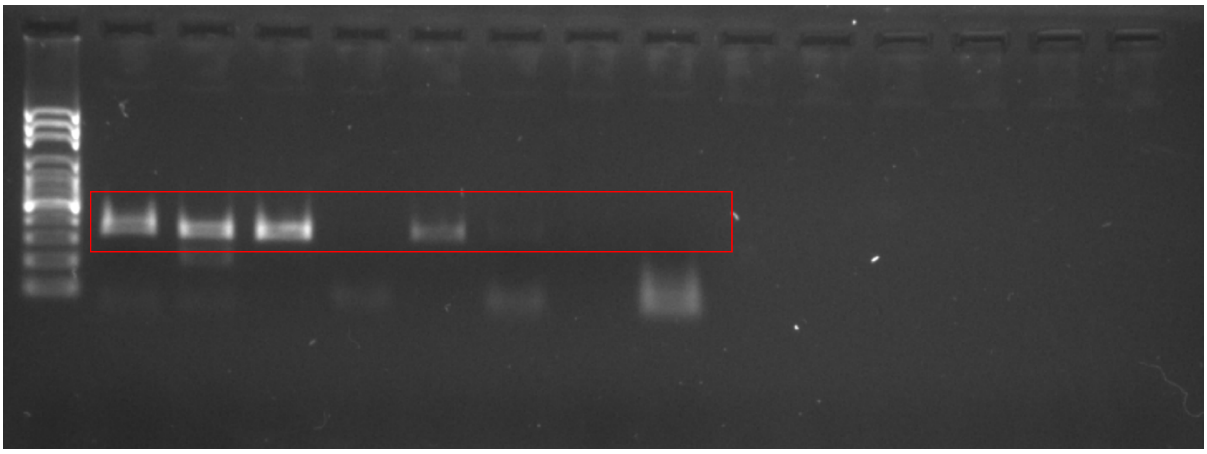

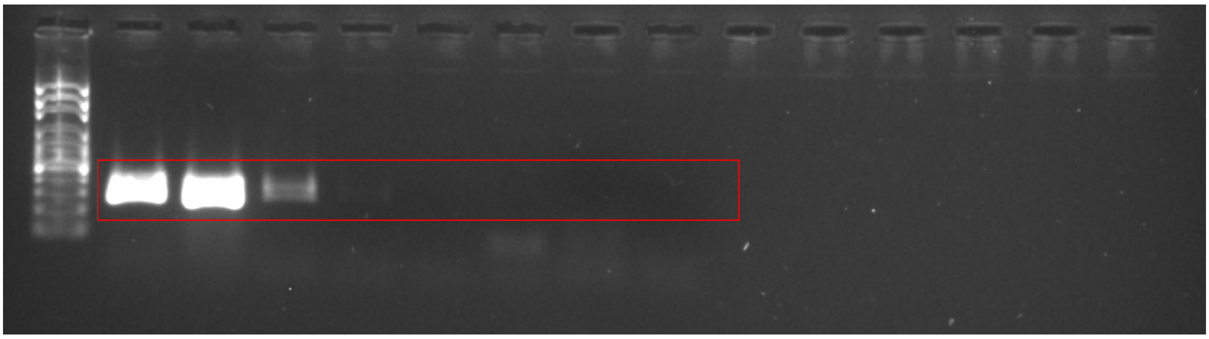

5.

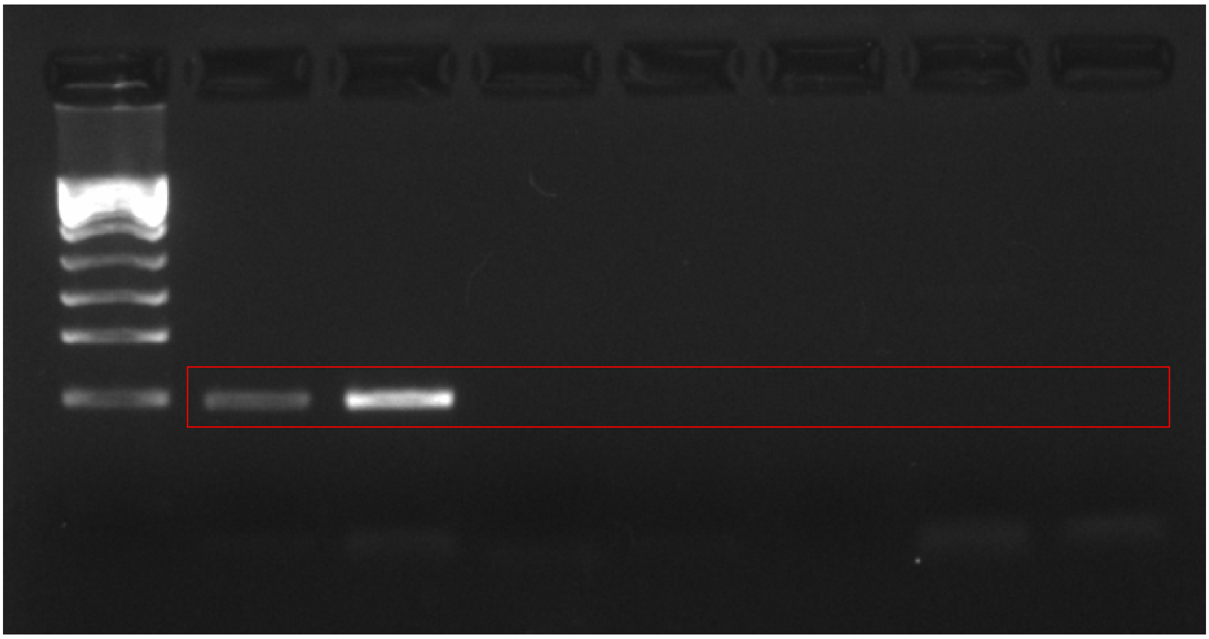

6.

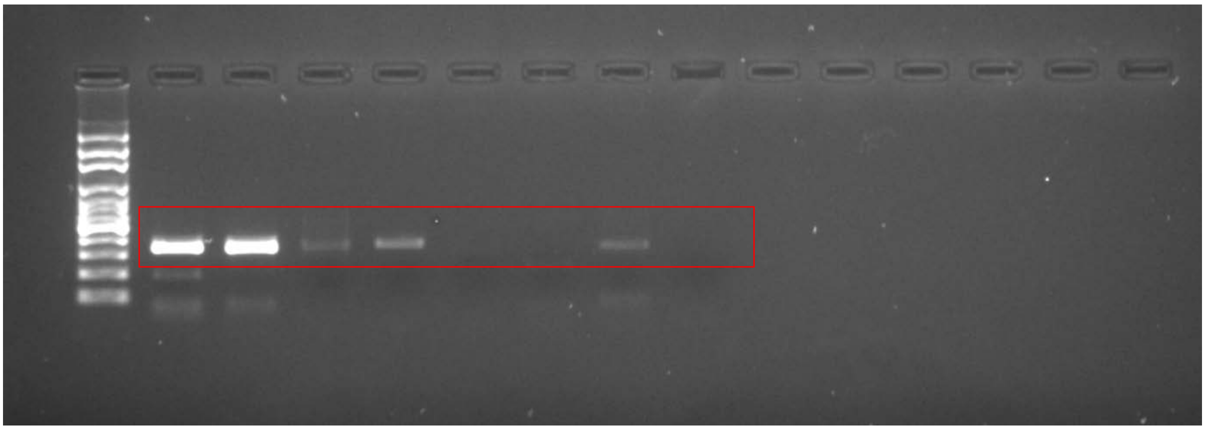

7.

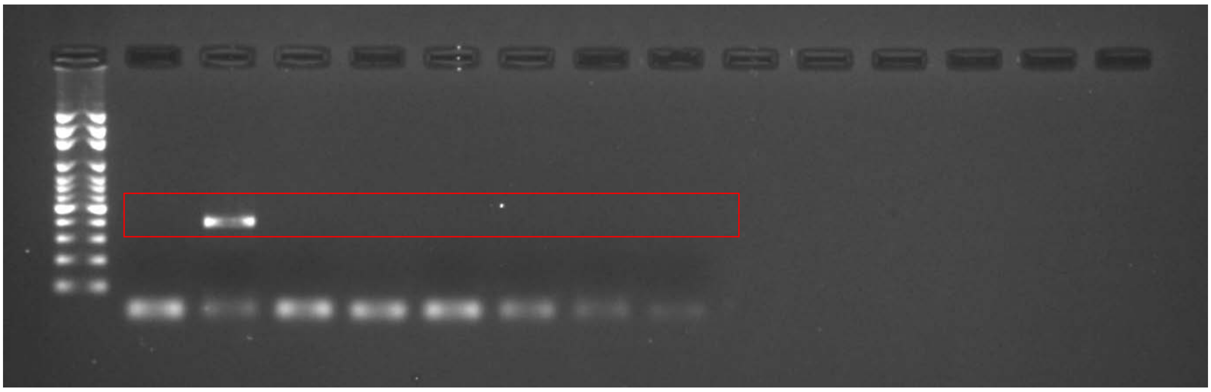
8.

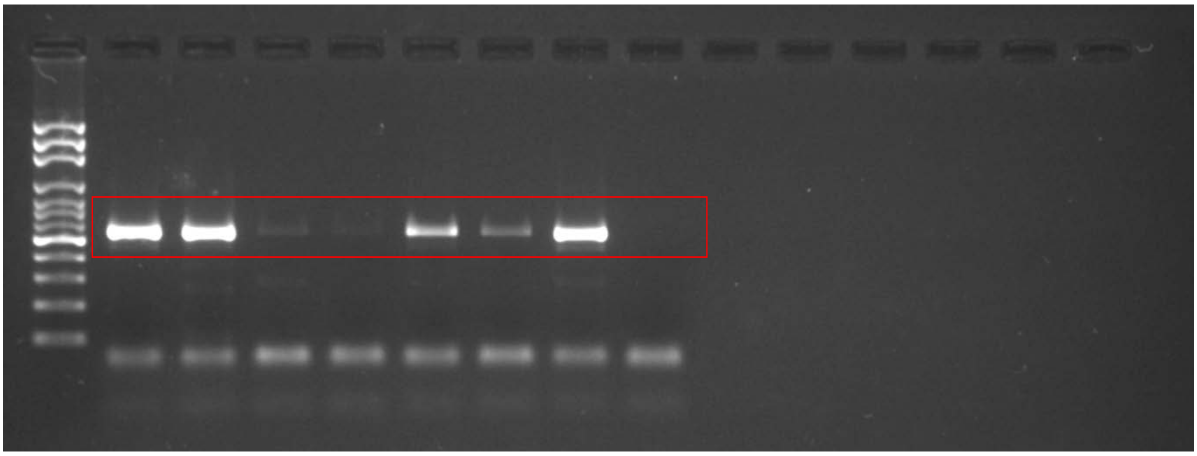
9.

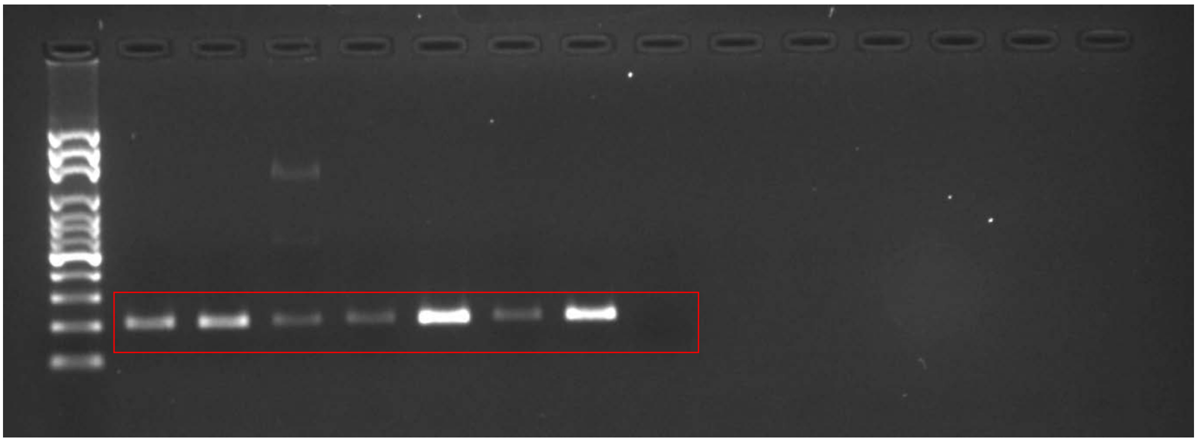
10.

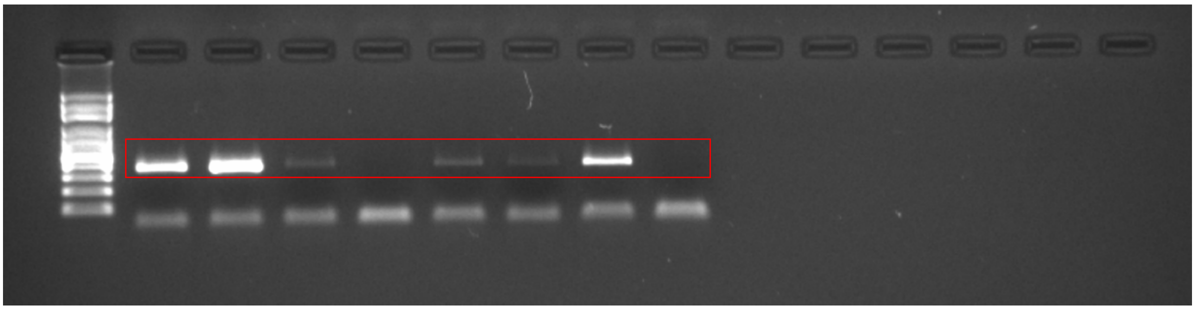
11.

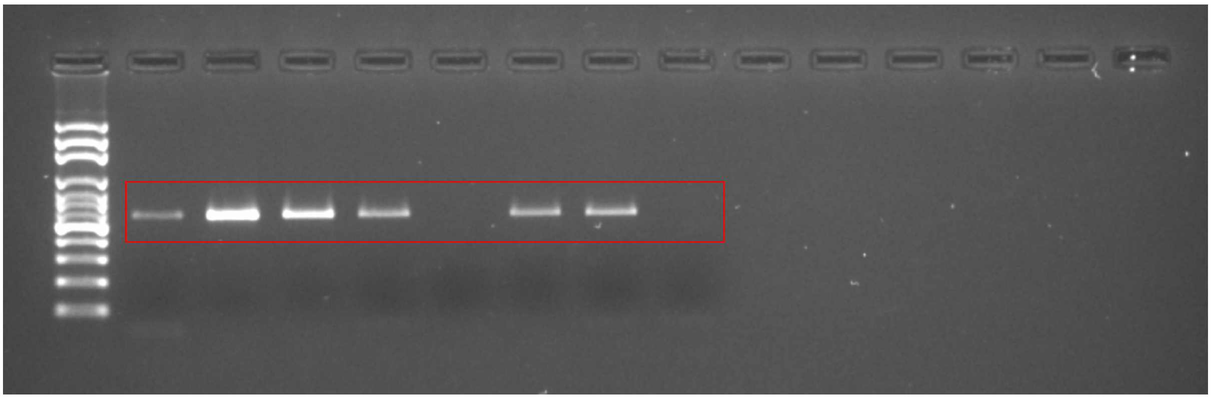

12.

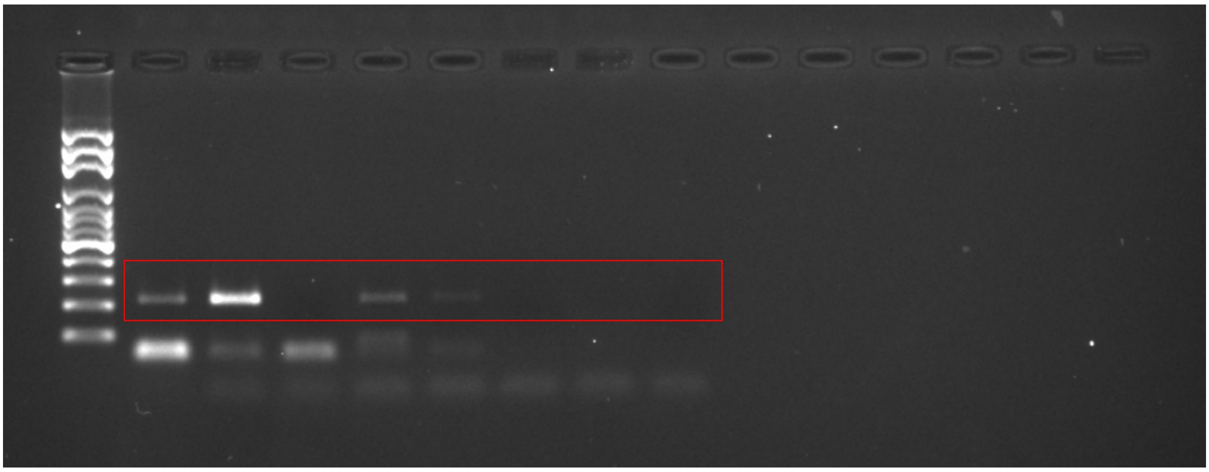

13.

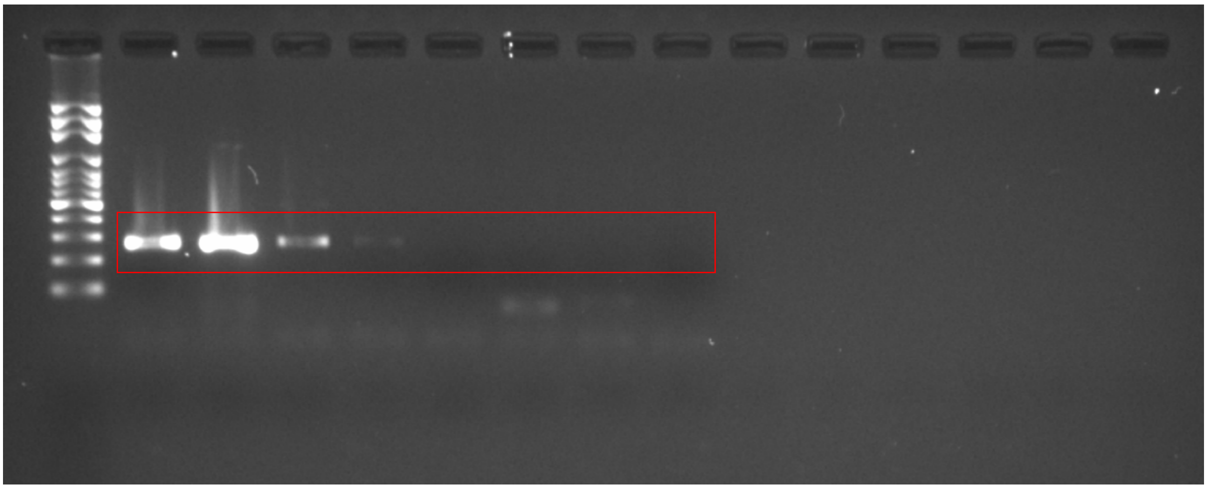

14.

15. 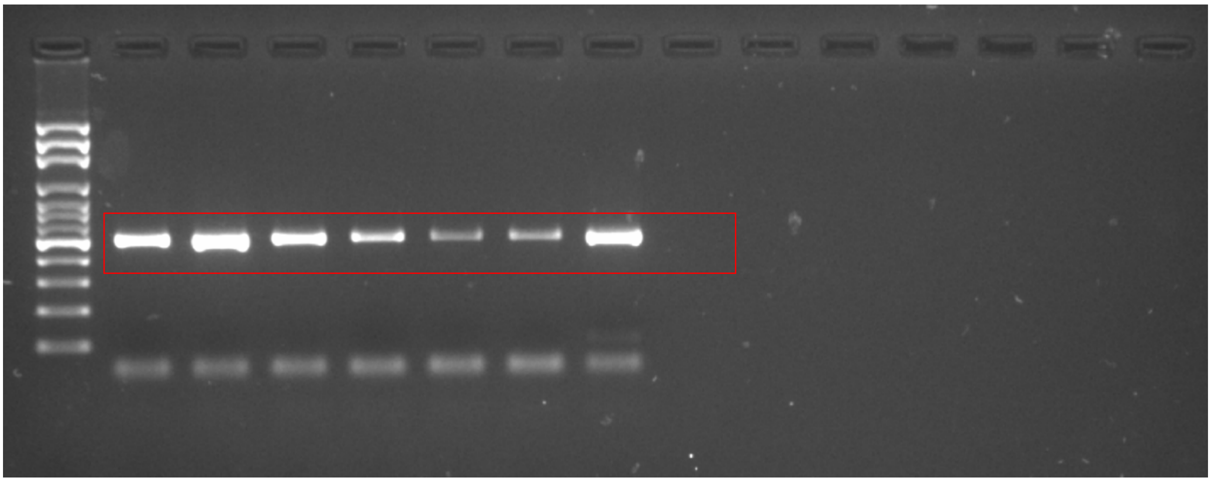

Supplement: Supplementary Information [file srep38658-s1.pdf]
